# Measuring climate knowledge: A systematic review of quantitative studies

**DOI:** 10.1016/j.isci.2025.111888

**Published:** 2025-01-25

**Authors:** Maruša Lubej, Žiga Petraš, Andrej Kirbiš

**Affiliations:** 1 University of Maribor Faculty of Arts, 2000 Maribor, Slovenia

**Keywords:** Earth sciences, Climatology, Environmental science, Education

## Abstract

Climate change is a pressing issue that necessitates a climate-literate population. This systematic literature review investigates how climate knowledge, a key component of climate literacy, is measured in scientific research. Analysis of 92 studies reveals that climate knowledge is primarily assessed as objective knowledge (facts and evidence). A minority of studies measured subjective knowledge. Most often, the target population was general adult population, followed by students and teachers. Furthermore, most studies are concentrated in North America and Europe, while climate knowledge remains underexplored in other regions. This review highlights the lack of consistency in the measurement of climate knowledge, particularly in defining its dimensions. Our findings underscore the need for future research to focus on developing a standardized, reliable, valid, and comprehensive instrument for measuring climate knowledge. This would enable the comparison of findings across different regions and populations.

## Graphical abstract

**Figure:**
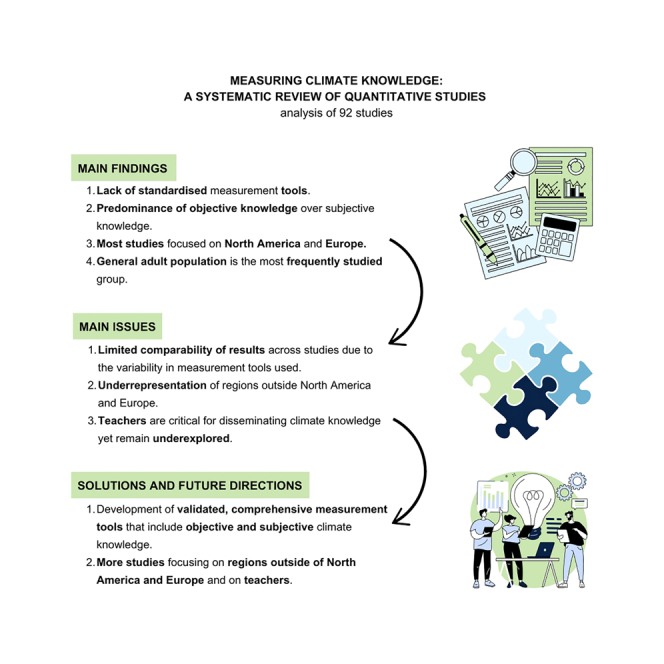

## Introduction

The global climate crisis, the most severe societal challenge of the 21st century, demands immediate intervention by our societies and economies. To address this problem, we need a climate-literate population. Climate-literate individuals understand the Earth’s climate, critically evaluate scientific knowledge about climate change, communicate effectively about it, and make informed climate-related decisions.^1^ Climate literacy is crucial for confronting climate change and can be seen as a combination of science, education, and policy Pan et al.^2^ However, despite the multidimensionality of climate literacy, most studies focus primarily on knowledge about climate change, which some even equate with climate literacy. Therefore, in this systematic literature review, we focus on climate knowledge as an integral component of climate literacy.

Researchers have explored various assessments of climate knowledge. For instance, a study by Baptiste^3^ examined knowledge about the causes of climate change among the general population in Jamaica. Andrea and Petkou^4^ examined knowledge about the greenhouse effect and the causes and effects of climate change among pre-primary and primary school teachers. Additionally, some studies use the measure of “polar knowledge” by Hamilton,^5^ which assesses knowledge of melting ice caps, sea level rise, and species threatened by climate change through multiple-choice questions. Some researchers admit that polar knowledge does not represent a comprehensive measurement of climate change knowledge.^6^ However, Hamilton^5^ acknowledges that polar knowledge relies on an understanding of basic climate facts. Further, other studies, such as those by Kolenatý et al.,^7^ evaluated three sub-dimensions of climate knowledge (system knowledge, action knowledge, and effectiveness knowledge) among elementary and secondary school students. Similarly, Yeh et al.^8^ examined climate literacy among Taiwanese public officials, focusing on three components of climate knowledge: issue, content, and strategic knowledge. Measurement approaches of climate knowledge often include structured survey instruments, such as Likert-type scales (e.g., DeWaters et al.^9^ and Powers et al.^10^), multiple-choice questions (e.g., Liu et al.^11^ and Boon^12^), and/or open-ended questions (e.g., McNeill^13^ and Vaughn and Bodzin et al.^14^). These examples show that researchers often examine climate knowledge through a multi-dimensional lens, with various dimensions that are interpreted differently across the literature.

While there is an existing systematic review regarding the measurement of negative emotional responses to climate change,^15^ no systematic review has specifically addressed how climate knowledge is measured. Climate knowledge encompasses subjective knowledge, defined as an individual’s self-reported knowledge that they believe is true (e.g., “*How much do you feel you know about global climate change?*”^10^), and objective knowledge, which refers to factual knowledge (e.g., *“What gas makes up most of the atmosphere?”*^16^). Although climate knowledge is a nuanced concept encompassing various domains such as knowledge of causes and consequences,^17^ its conceptualization is rarely the focus of research. A comprehensive review of climate knowledge measurement is therefore needed.^18^

Therefore, the primary aim of our systematic literature review is to fill this gap by investigating how climate knowledge is measured in scientific research. We also provide recommendations for developing standardized, validated indicators of climate knowledge. The lack of standardized climate knowledge indicators has been emphasized by previous researchers (e.g., Kolenatý et al.^7^ and Kutywayo et al.^19^). Furthermore, higher levels of both subjective and objective knowledge strengthen people’s beliefs about climate change, highlighting the importance of considering both objective and subjective knowledge.^20^ Objective climate knowledge is the more prevalent measure of the two.^21^

This paper aims to analyze climate knowledge measurement instruments and determine how researchers examine climate knowledge (objective and subjective knowledge). We aim to answer the following research questions.

(1) What survey instruments are used to measure climate knowledge?
(2) Which dimensions are included in measuring climate knowledge?
(3) What types of knowledge are prevalent in the measure of climate knowledge—objective or subjective knowledge?

We begin this systematic review by outlining our strategy to identify the relevant studies included in our sample. Our approach included a comprehensive database search and the selection of articles that met our inclusion criteria. The results section provides a descriptive analysis focusing on the measurement of climate knowledge and its dimensions. We conclude the systematic literature review by summarizing key insights and proposing areas for future research.

## Method

### Design and search strategy

While conducting this systematic review, we followed the preferred reporting items for systematic reviews and meta-analysis (PRISMA) guidelines.^22^ The initial search for articles on climate knowledge was conducted in March 2024 using four databases: Web of Science, Scopus, ERIC, and Academic Search Complete. We focused our search on articles published from 2010 onwards to ensure that the review includes the most recent and relevant studies. This decision was further supported by the observation that the number of relevant studies published before 2010 dropped substantially in our initial search across the mentioned databases. Articles included in the review were written in English. Following Kranz et al.,^23^ we used the following search terms to identify relevant articles:

“climate literacy” OR “climate change literacy” OR “climate science literacy” OR “environmental literacy” OR “climate knowledge” OR “climate science knowledge” OR “climate change knowledge” OR “environmental knowledge”

### Inclusion and exclusion criteria

**Figure 1:**
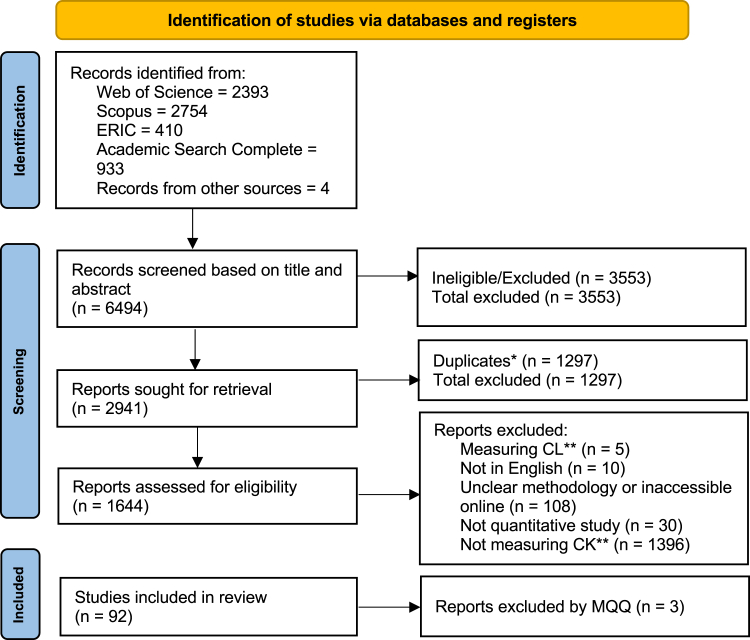
PRISMA text selection process ∗Duplicates assessed after first screening of abstract and title. ∗∗CL, climate literacy; CK, climate knowledge.

We conducted a two-step selection process (Figure 1). We first screened the articles and abstracts. Then, full texts were retrieved for the second screening. Studies were excluded if they focused on climate literacy and not knowledge (5), if they did not indicate measurement of climate knowledge (1,396), if they were not written in English (10) and if the methodology was unclear (e.g., how climate knowledge was measured) or inaccessible online (108). Additionally, qualitative studies (30) were excluded. We contacted the authors of the studies that were not accessible, which resulted in the retrieval of five studies. As detailed below, after quality assessment, three additional studies were excluded due to low scores. The final dataset for analysis comprised 92 studies (see List S4 for the full list).

#### Extraction and analysis

Data for the included studies were extracted, analyzed, and compared using MS Excel summary tables (Palmaccio et al. and Berman et al.^24^^,^^25^). We retrieved several details regarding the studies, including authors and year of publication, title, country, sample size, and age(s) of the study population. Additionally, we collected data on the study design, instrument used to measure climate knowledge and which dimensions of climate knowledge were covered in each included paper.

While the differences in survey questions limit comparability across studies, we focused on identifying overarching themes and dimensions common to different measurement instruments. This approach allowed us to form meaningful synthesis by categorizing the different climate knowledge measurement domains and approaches (e.g., into constructs such as objective and subjective knowledge).

#### Quality assessment

To assess the methodological quality of the included studies, a modified version of the methodological quality questionnaire (MQQ) was used (see Risko et al. and Scott et al.^26^^,^^27^). The adapted MQQ includes seven key indicators designed to evaluate the overall quality and effectiveness of the research: (1) alignment with relevant theoretical frameworks, (2) study objectives that can be empirically investigated, (3) clarity of included methods, (4) measurement reliability, (5) measurement validity, (6) detailed description of study population, and (7) consistency between findings and collected data (Scott et al., 2008).

The MQQ employs a scoring system where each quality indicator receives a point between 1 (lowest) and 7 (highest). Scott et al.^27^ assessed studies according to quality criteria and categorized studies in three groups: (1) studies meeting all criteria, (2) re-evaluating studies scoring 4–6 for potential inclusion, and (3) excluding studies scoring 1–3. We conducted a similar process, using the MQQ scoring system, the three excluding studies scoring 1–3. The quality assessment of the studies in our final sample is presented in Table S1.

## Results

### Descriptive analysis

**Figure 2:**
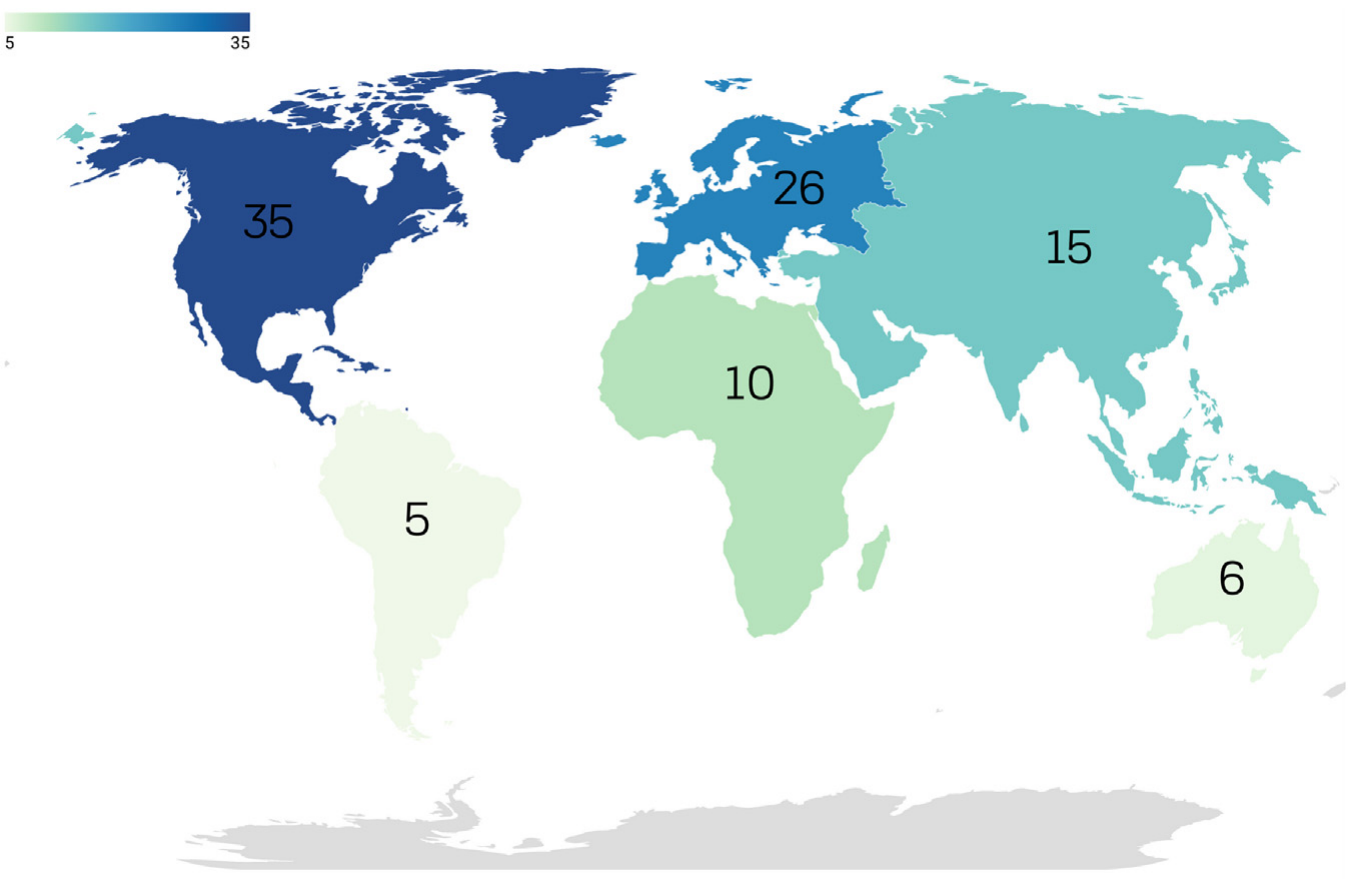
Number of studies by continent Some of the studies included multiple continents (e.g., Rooney-Varga et al.^28^ conducted a study among participants from North and South America, Europe, and Africa).

This section provides a descriptive analysis of the included studies. The results are organized by geographical distribution, sample size and data sources, methodological approaches, and target populations. Most studies in our sample (*n* = 35) were conducted in North America, followed by European countries (*n* = 26), Asia (*n* = 15), Africa (*n* = 10), Australia (*n* = 6), and South America (*n* = 5) (Figure 2).

Total sample sizes ranged widely from 55 to 45,823 participants (Table S2). The nationally representative data used in the included studies were gathered from various sources. Two studies were conducted with the data from Afrobarometer,^29^^,^^30^ two from Australian Survey of Social Attitudes (AuSSA) from 2017 to 2018.^6^^,^^31^ One study used data from the Social Futures and Life Pathways (“Our Lives”) project,^6^ and one from Gallup poll.^32^ Afrobarometer surveys were conducted within the seventh round with specific questions about climate change, including 45,823 interviews in 34 countries.^30^ The Australian Survey of Social Attitudes from 2017 (AuSSA) is a four-wave national social survey with a representative sample of the Australian adult population and a response rate of 28%.^6^ Additionally, Tranter et al.^33^ also analyzed the data from 2019 Our Lives survey of young people from the state of Queensland (QLD), Australia. This is a longitudinal study in which youth have been surveyed every 2 to 3 years from age 13 in 2006 to age 26 in 2019. The data for the research derives from the seventh wave of Our Lives Survey, which replicated the climate attitudes and knowledge questions from the 2018 AuSSA. The survey was completed by 1593 participants.

The studies employed a range of methodologies to assess climate change knowledge, including cross-sectional (*n* = 61), experimental (*n* = 14) designs, and case studies (*n* = 4). Experimental studies included pre-tests and post-tests to gather data before and after interventions, and these were frequently conducted in classroom settings (e.g., Flora et al.^34^; Nussbaum et al.^35^; Asshoff et al.^36^; and Fernandez et al.^37^). Among the case studies, Bedford^38^ examined how increased knowledge about the climate system might influence opinions on global warming among 458 students at a primarily undergraduate university in the US. Additionally, Siegner and Stapert^39^ analyzed the effects of implementing a climate change curriculum on elementary school students. In another study, DeCamp^40^ researched students’ basic knowledge of climate change and how it changed after the implementation of climate modules. Adu-Boateng et al.^41^ study was both cross-sectional and a case study, examining existing nature-based solutions to climate change, perceptions about climate, and strategies to implement new nature-based solutions.

Two studies employed a longitudinal design and nine of them adopted a cross-country approach. Taddicken^21^ collected data from 935 respondents across three waves in Germany from 2013 to 2014, assessing people’s knowledge, attitudes, and media use related to climate change. Boon^12^ conducted a study on 87 pre-service teachers aged 17 to 26, examining the development of their climate change attitudes over time. Examples of cross-country studies include Dijkstra and Goedhart,^42^ Geiger et al.,^43^ Harker-Schuch et al.,^44^ and Alenda-Demoutiez.^30^ For instance, Nepras et al.^18^ focused on students’ knowledge and attitudes about climate change across the Czech Republic, the United Kingdom, and Portugal.

Some studies (*n* = 60) in our sample focused on developing and validating instruments to measure attitudes or knowledge about climate change. For example, Dijkstra and Goedhart^42^ developed a 63-item questionnaire attitudes toward climate change and science instrument (ACSI). Additionally, Walker and McNeal^45^ developed the climate stewardship survey (CSS) to measure knowledge and perceptions about climate change (see Table S2).

**Figure 3:**
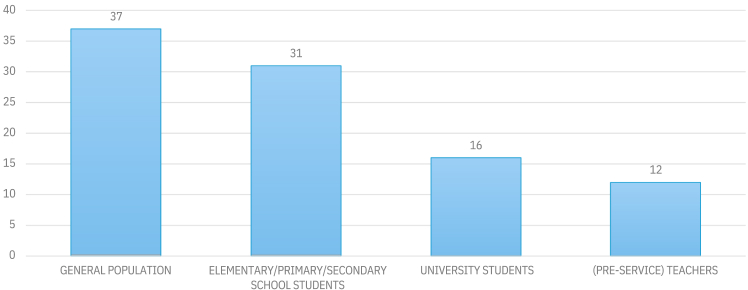
Number of studies by target population Some studies included multiple samples from different population groups (e.g., Schollaert Uz et al.^50^ examined three samples: fifth graders, sixth graders, and teachers).

Most of the studies in our sample were conducted among the general adult population (*n* = 37), followed by studies involving students at elementary, primary, middle, and secondary school levels (*N* = 29). Furthermore, 16 studies focused on university students, while a minority (*n* = 12) investigated teachers (Figure 3). The studies that focused on teachers included educators from various disciplines and levels. For example, Anyanwu and Le Grange^46^ studied 194 high school geography teachers, Asshoff et al.^36^ included 110 pre-service biology teachers, Boon^12^ focused on 87 Australian pre-service teachers, Borhan and Ismail^47^ on 173 in Malaysia and Fernandez et al.^37^ on 102 in Spain. Among the studies that gathered data on students, adults, and teachers, three studies^11^^,^^48^^,^^49^ specifically focused on farmers. Abunyewah et al.^49^ collected data from 874 farmers aged 18 to over 63 years, Das et al.^48^ conducted their study among 200 adult farmers in India, and Liu et al.^11^ surveyed 481 farmers in the USA.

In summary, the studies in our sample varied significantly in terms of geographical distribution, methodological approaches, data sources, and target populations. However, notable gaps remain in geographical representation and study design. For example, the majority of the studies were conducted in North America and Europe, which may limit the generalizability of findings to other regions. Similarly, there is a predominance of cross-sectional designs. More longitudinal studies are needed to provide deeper insights into temporal changes.

### Measurement of climate knowledge

#### Types of survey instruments

The number of items (“items” refers to individual survey questions systematically measuring various aspects of climate knowledge) in the studies reviewed ranged from 2 to 81 items (see Table S3). For instance, Aruta^51^ used only two items “*explain how carbon-dioxide emissions affect global climate change*” and “*explain why some countries suffer more from global climate change than others*” on a four-point scale ranging from 1 (I couldn’t do this) to 4 (I could do this easily) to assess climate change knowledge. The study by Bozoglu et al.^52^ used 40 items for measuring climate change knowledge on a scale from strongly disagree (1) to strongly agree (5). The summation score was transformed into total knowledge score and divided in three groups: low knowledge level (lower than 2.5), moderate knowledge level (range from 2.5 to 3.5) and high knowledge level (score higher than 3.5). Two studies were conducted with more than 70 items. DeCamp^40^ examined “content” climate change knowledge among US university students at a Midwestern university with 76 items in three sections: climate mitigation, adaptation section and climate justice section. While Gutierrez et al.^53^ examined objective knowledge with 76 items, grouped to represent the seven “essential principles of climate science”, the responses were coded as either correct (1) or incorrect (0). This variation in the number of items across studies may influence the depth of knowledge assessed.

Most instruments used in included studies to assess climate knowledge were adapted from existing measures. The most used and frequently modified measure was developed by Tobler^17^ in their study of Swiss public’s understanding of climate change^21^^,^^37^^,^^49^^,^^54^^,^^55^^,^^56^^,^^57^^,^^58^^,^^59^ (*n* = 9). The instrument validated by Tobler et al.^17^ measures four domains of climate knowledge, with example items including “*Carbon dioxide (CO2) is a greenhouse gas”* for the physical knowledge domain, *“The increase of greenhouse gases is mainly caused by human activities”* for the causes of climate change domain, *“For the next few decades, the majority of climate scientists expect … a warmer climate to increase water evaporation, which will lead to an overall decrease of the sea level”* for the expected consequences domain, and *“A large part of CO2 emissions in Switzerland is produced by heating”* for the action-related knowledge domain. Additionally, many studies used the Yale climate change communication survey^39^^,^^28^^,^^53^^,^^60^^,^^61^^,^^62^ (*n* = 7). While less frequent, some studies employed instruments by Boyes et al.^63^ or the “polar” knowledge by Hamilton,^5^ which was also used in three subsequent studies.^6^^,^^31^^,^^33^

Some studies (*n* = 7) constructed their own instruments to assess climate change knowledge (e.g., Hallar et al.^16^; Walker and McNeal^45^; Schollaert Uz et al.^50^; Huxster et al.^64^; and Nussbaum et al.^35^). For instance, Gazzaz and Aldeseet^65^ developed a 30-item climate change knowledge test (CCKT) specifically designed for their study. The CCKT assessed three types of knowledge: about the nature, causes, and effects of climate change, each consisting of ten items. Similarly, Higuchi et al.^66^ created a 15-item instrument to assess knowledge about various climate change domains, including carbon, consumption patterns, landslides, and transportation.

Many studies (*n* = 27) divided questions in different sections, which often included causes, impacts/consequences, characteristics and solutions to climate change (e.g., Borhan and Ismail^47^; Anyanwu and Le Grange^46^; Asshoff et al.^36^; Connor et al.^57^; Abunyewah et al.^49^; Asgarizadeh et al.^58^; DeCamp^40^). For instance, Alenda-Demoutiez^30^ measured three items, one for each dimension: “*What about you, which of the following do you think is the main cause of climate change, or haven’t you heard enough to say?”, “Do you think climate change is making life in [country] better or worse, or haven’t you heard enough to say?”* and “*Do you think that climate change needs to be stopped? [If yes] How much do you think that ordinary Ghanaians can do to stop climate change?”*.

Among the studies that used Likert agreement scales (*n* = 18) to measure climate change knowledge, the most frequently covered topics were related to the physical science concepts related to climate change, particularly the role of greenhouse gases, the greenhouse effect, and carbon dioxide (e.g., Taddicken et al.^21^; Garcia-Vinuesa et al.^67^; Kolenaty et al.^68^). Examples of items include: “*The best way to deal with climate change would be to reduce or eliminate carbon-based fuel sources*,” “*The greenhouse effect will not bring changes in global food production*,” “*If we stop emitting greenhouse gases, we will not be affected by climate change*,” “*CO2 is the main gas responsible for climate change*,” and “*Carbon dioxide (CO2) is a greenhouse gas*”^11^^,^^18^^,^^21^^,^^49^^,^^64^^,^^67^^,^^68^^,^^69^^,^^70^^,^^71^^,^^72^^,^^73^^,^^74^^,^^75^^,^^76^^,^^77^ (see Table S3).

Some studies measured climate change knowledge using correct/incorrect (*n* = 9) or true/false (*n* = 20) statements, with varying numbers of questions. The most common topics covered were related to physical sciences, including concepts such as CO2, temperature, the greenhouse effect, and the ozone layer (e.g., Borhan and Ismail^47^; Walker and McNeal^45^; Flora et al; ^34^; Stevenson et al; ^54^; Stevenson et al; ^55^; Higuchi et al; ^66^; Escoz Roldan et al; ^78^; Rahman et al.^79^; Thaller and Brudermann^80^; Asshoff et al.^36^; Tremoliere and Djeriouat^56^; Das et al.^48^; Bozoglu et al.^52^; Wang et al.^81^; Bedford^38^; Asgarizadeh et al.^58^; Fernandez et al.^37^). Mumpower et al.^82^ used 10 true/false statements, for example: “*The major cause of increased atmospheric concentration of greenhouse gases is human burning of fossil fuels*,” “*Nitrous oxide is a greenhouse gas*,” *and* “*Aerosols are airborne particles that are known to contribute to the formation of clouds and precipitation*”*.* Similarly, Hu et al.^83^ used 8 statements, including: “*Burning oil produces CO2*,” “*CO2 is harmful to plants*,” “*Nuclear power plants emit CO2 during operation*,” and “*At the same quantity, CO2 is more harmful to the climate than methane.*” (see Table S3).

Multiple-choice questions were used in 29 studies in our sample. The most common questions covered the causes and consequences of climate change. Examples of such questions include: “*According to climate scientists, how has the amount of carbon dioxide in the atmosphere changed since the start of the Industrial Revolution 150 years ago?*”; *“What do you know about the causes and impacts of floods?*”; “*What effect does the greenhouse effect describe?*”; “*Carbon dioxide is responsible for approximately …* ”.^6^^,^^8^^,^^13^^,^^14^^,^^31^^,^^33^^,^^35^^,^^39^^,^^40^^,^^44^^,^^60^^,^^61^^,^^50^^,^^81^^,^^84^^,^^85^^,^^86^^,^^87^^,^^88^

Across the studies, climate change knowledge was measured in different ways with different types and numbers of questions. However, the core objective remained consistent across the included studies: to measure knowledge about climate change, whether in relation to its causes, consequences, impacts, solutions, or a combination of these. The majority of questions in the measurement instruments used in the studies from our sample focused on objective knowledge, while subjective knowledge was addressed to a lesser extent. The most frequently addressed topics were the causes of climate change and potential preventive actions.

#### Construct operationalization

As mentioned earlier, climate knowledge can be measured as subjective (self-reported) and objective (actual), and both types should be considered^20^^,^^21^^,^^89^ as both shape climate attitudes and behaviors.^20^ Out of all studies in our sample that assessed objective climate knowledge (*n* = 89), 29 used true/false or correct/incorrect statements (e.g., Borhan and Ismail^47^; Dijkstra and Goedhart^42^; Bedford;^38^ Bremer and Linnenluecke^90^; Escoz Roldán et al.^78^; Bozoglu et al.^52^; Asgarizadeh et al.^58^; and Fernández et al.^37^). On the other hand, studies that incorporated subjective knowledge of climate change measures (*n* = 26) used varying approaches (see Table S3). For example, some researchers examined how much the respondent feels they know about or are informed about climate change (e.g., DeWaters et al.^9^). Others focused on how certain the respondent is about the accuracy of their knowledge (e.g., Thaller and Bruderman^80^ and Fischer & Said^91^) or how confident they are in their own knowledge (Nyarko and Petcovic^92^).

The majority of the studies in our sample assessed only objective knowledge (*n* = 66), 23 studies measured both subjective and objective knowledge (e.g., DeWaters et al.^9^; Karpudewan et al.^93^; Huxster et al.^64^; Fischer and Said^91^; García-Vinuesa et al.^67^; Gutierrez et al.^53^) and three studies relied solely on subjective knowledge measures.^51^^,^^94^^,^^95^ For instance, Vainio and Paloniemi^94^ assessed climate change knowledge through self-reported understanding using three items, focusing on the causes, consequences, and solutions to climate change. These included: “*Personally, do you think that you are well informed or not about … the different causes of climate change?*”, “… *the different consequences of climate change?*” and “… *ways in which we can fight climate change?*” This was measured on a four-point scale from 1 (not informed at all) to 4 (very well informed). Additionally, we found that three studies^41^^,^^51^^,^^96^ did not explicitly state that they examined subjective measures of climate knowledge.

Most studies that measured subjective knowledge also included objective knowledge (*n* = 23) assessment (e.g., McCright^32^; DeWaters et al.^9^; Huxster et al.^64^; Fischer and Said^91^; and García-Vinuesa et al.^67^). Five studies incorporated items gauging participants’ certainty of the correctness of their objective knowledge measure (e.g., Thaller and Bruderman^80^ and Fischer and Said^91^) and their confidence in the accuracy of their objective knowledge (e.g., Nyarko and Petcovic,^92^ 2021 and Trémolière and Djeriouat^56^). For instance, the German study by Fischer and Said^91^ asked participants “*How certain are you that your answer is correct?*” on a 6-point scale ranging from 50% (not at all certain, I was guessing) to 100% (certain, I know the answer). Similarly, the study by Trémolière and Djeriouat,^56^ conducted with participants from the United States, required them to indicate their confidence (0 – not at all confident, to 100 – totally confident) after answering each objective knowledge item. A higher confidence level than the percentage of correct answers suggests overconfidence, while a lower confidence level indicates underconfidence. No difference between the two suggests no bias. In the study by Karpudewan et al.,^93^ participants were asked, “*Are you sure about your answer given to the previous two questions?”* with “yes” or “no” as the response options.

Of the studies measuring objective knowledge, 18 used a Likert agreement/disagreement scale (e.g., Nepras et al.^18^ and Thacker^77^), and 9 used correct/incorrect answer formats (e.g., Asgarizadeh et al.^58^; Fernandez et al.^37^). Additionally, 20 studies used true/false statements (e.g., Fischer and Said^91^; Hurst Loo and Walker^59^), 6 relied on open-ended questions (e.g., McNeill & Vaughn^13^; Jurek et al.^97^), and 29 used multiple-choice questions (e.g., Boon^12^; Chuvieco et al.^98^) (see Table S3).

In multiple-choice questions that measured objective knowledge, most of the studies provided 4 or 5 response options about causes, impacts and solutions to climate change (e.g., Hallar et al.^16^; Javeline et al.^99^; Klapp and Bouvier-Brown^61^). Additionally, 6 studies measured objective knowledge using open-ended questions (McNeill and Vaughn^13^; Bodzin et al.^14^; Schollaert Uz et al.^50^; Karpudewan and Mohd Ali Khan^100^; Jurek et al.^97^; Kolenaty et al.^68^). Examples of such questions include: “*State the factors which cause climate change*”^97^ and “*What would it actually take for all the people on our planet to lower the levels of carbon dioxide in the atmosphere?*”*.*^14^ Further examples include: “*What is the name of the microscopic plant in the ocean that forms the base of the food web and creates half of all the oxygen we breathe?*”,^50^ and “*What are three human behaviors that impact climate change? Why?*”*.*^13^ Similar to all the studies in our sample that assessed objective knowledge, they primarily focused on measuring general knowledge, causes, consequences and impacts of climate change.

Some studies used yes/no answers to measure objective knowledge (*n* = 6) (e.g., Ebuehi and Olusanya^101^; Rooney-Varga et al.^28^; Helbling et al.^29^; Rooney-Varga et al.^102^). Others used true/false statements (e.g., Hu et al.^83^; Thaller and Brudermann^80^; Gazzaz and Aldeseet^65^) (*n* = 20). Both sets of studies mostly relied on questions about causes of climate change (see Table S3). Among the studies using true/false formats, two employed a different set of response options. In Meira-Cartea et al.,^103^ the response options ranged from 1 (absolutely true) to 4 (absolutely false). In Walker and McNeal,^45^ the options ranged from 1 (definitely false) to 4 (definitely true), whereas other studies opted for a simpler true/false format. Similarly to these formats, some studies (*n* = 10) used correct/incorrect questions to measure objective knowledge (e.g., Flora et al.^34^; Das et al.^48^; Gurierrez et al.^53^; Asgarizadeh et al.^58^).

The studies adopted different approaches to operationalize climate knowledge, using objective, subjective, or both types of measures. Studies that focused on objective knowledge used different formats, including Likert scales, open-ended questions, multiple-choice questions, and true/false statements. Subjective knowledge assessments were less common and included measures of self-reported understanding and confidence in one’s knowledge. The variability in operationalization limits the comparability of results across studies.

#### Dimensions of climate knowledge

The studies in our review operationalized the dimensions of climate knowledge in various ways, focusing on different aspects. We categorized the studies into four groups based on the dimensions of climate knowledge they measured: (1) studies that did not explicitly state the multidimensionality of their instrument (*n* = 55); (2) studies that examined two dimensions (*n* = 4); (3) studies that examined three dimensions (*n* = 24); and (4) studies that examined more than three dimensions (*n* = 9). We define dimensions as distinct categories of climate knowledge that capture specific aspects of climate knowledge as operationalized by the studies included in our sample, such as knowledge of climatic physical features, causal factors, and the impacts of climate change.

Fewer than half of the studies explicitly stated that they examined multiple dimensions (three or more) (*n* = 33) (e.g., Borhan and Ismail^47^; Walker and McNeal;^45^ Anyanwu and Le Grange^46^; Asshoff et al.^36^; Alenda-Demoutiez^30^; Yeh et al.^8^). However, among all the studies measuring climate knowledge with multiple dimensions (*n* = 37), the most common division was into three dimensions (*n* = 23). For instance, Powers et al.^10^ measured climate literacy with the following dimensions: climate science (e.g., “*CO2 is the greenhouse gas we are most concerned about limiting emissions of, to reduce global warming*”), causes and effects of climate change (e.g., “*Fossil fuel combustion is a cause of climate change*”), and climate change mitigation strategies (e.g., “*Recycling more*” will help reduce or slow down climate change) (see Table S3). Additionally, Abunyewah et al.^49^ conducted a study among farmers in Ghana, examining three dimensions of climate change knowledge: causes, physical characteristics, and consequences of climate change.

Other operationalizations of climate knowledge dimensions vary widely. For example, Geiger et al.^43^ examined three types of environmental knowledge (system knowledge, action-related knowledge, and efficiency knowledge) among the general populations in Argentina and Colombia. Each knowledge type included 12 items across several domains, such as climate change, energy, food, health, water, pollution, and waste. A similar approach was used by Kolenatý et al.^7^ and Player et al.,^87^ who examined three key dimensions of climate change knowledge: system, action, and effectiveness knowledge. Additionally, Acevedo et al.^74^ divided environmental knowledge into three categories: air and soil (e.g., “*You believe that there is a decrease in livestock and pasture planting*”), biodiversity (e.g., “*Do you think it is necessary to protect biodiversity*”), and climate change (e.g., “*You believe that climate change can be made to slow down*”), using a scale from strongly agree (1) to strongly disagree (5).

Only a minority of the studies examined climate knowledge with four dimensions (*n* = 6) (e.g., Escoz Roldán et al.^78^; Lin and Wang^76^; Kurowski et al.^62^; Harker-Schuch^44^). For instance, Meira-Cartea et al.^103^ conducted a study among Spanish undergraduate students, examining four dimensions of climate knowledge: causes, consequences, biophysical processes, and actions, responses, and solutions to climate change. The same dimensions were used by Escoz Roldán et al.,^78^ while Asgarizadeh^58^ examined physical knowledge, causes, consequences, and action-related knowledge. Additionally, Pan et al.^2^ used 30 items that were grouped into four categories: causes, consequences, human engagement, and general physical knowledge. These items were measured using a true-false response format.

Even fewer studies used a five-dimensional approach to measure climate knowledge (*n* = 3) (e.g., Lin and Wang^76^). For instance, in a longitudinal study, Taddicken et al.^21^ covered five domains of climate change knowledge, adapted from Tobler et al.^17^: causal knowledge (climate change and causes), basic knowledge (physical knowledge about CO_2_ and the greenhouse effect), effects knowledge (expected consequences of climate change), and action-related knowledge. The same study also included procedural knowledge (understanding how scientific findings are developed in climate research), a dimension previously used by Miller,^104^ Nisbet et al.,^105^ Bauer et al.,^106^ and Taddicken and Reif.^107^ This approach, based on Taddicken et al.,^21^ was also used by Lin and Wang^76^ in a Taiwanese study. Additionally, DeWaters et al.^9^ used the following categories: climate science, causes and impacts of climate change, mitigating climate change, general knowledge, and self-assessment. These were measured using multiple-choice questions.

**Figure 4:**
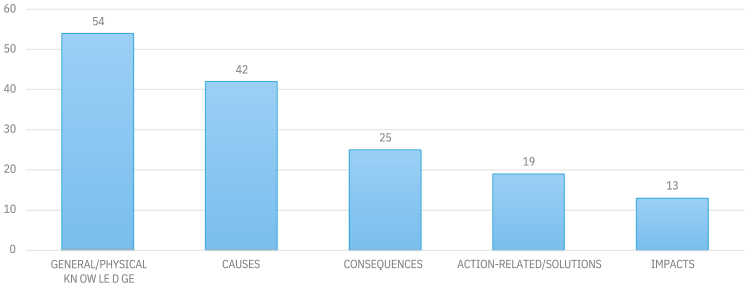
Most common dimensions of climate change knowledge measurement

As shown in Figure 4, the most common dimension in the studies in our sample was general/physical knowledge (*n* = 54), which was measured with questions like “*Which of the following is NOT a greenhouse gas?*”^85^ and “*What gas makes up the most of the atmosphere?*”.^16^ The next most frequently assessed dimensions were the causes of climate change (*n* = 42), with items like “*Burning releases greenhouse gasses, causing the global warming*”^92^ and “*Deforestation is one of the causes of climate change.*”.^65^ The third most commonly assessed dimension was knowledge of the consequences of climate change (*n* = 25), measured with statements like “*An increasing amount of greenhouse gases increases the risk of more UV-radiation and therefore a larger risk of skin cancer*”,^108^ “*Climate change will have consequences for the nature and human lives*”,^2^ and *“The climate change will increase the number of earthquakes and tsunamis*”.^78^ Other dimensions, such as impacts, action-related knowledge, and solutions to climate change, were used to a lesser extent than these categories.

It is evident that studies in our sample used varying measuring approaches for climate knowledge assessment. Less than half of the studies explicitly addressed a multidimensional approach to measuring knowledge. Among the studies that used multiple dimensions to operationalize climate knowledge, the majority included three dimensions. General and physical knowledge were the most frequently assessed dimensions across the studies. As noted by Asshoff et al.,^36^ existing measurement instruments capture climate knowledge to a limited extent, prompting them to develop a new tool for their study. This limitation also motivated other researchers to create their own measurement instruments, often in the form of multiple-choice questions, to gain a deeper understanding of respondents’ climate knowledge. Several studies confirmed the reliability of their newly developed instruments through Cronbach’s alpha (e.g., McCright^32^; Flora et al;^34^; Kolenaty et al; ^68^; Jama et al; ^75^; Lin and Wang; ^76^ Nepras et al; ^18^).

### Future research directions

Several included studies mention methodological limitations, such as nonrepresentative samples, which limits the generalizability of their findings (*n* = 12) (e.g., Bodzin et al.^14^; Geiger et al.^43^; Hu et al.^83^; Rahman et al.^79^; Asshoff et al.^36^; Klapp and Bouvier-Brown^61^; Nyarko and Petcovic^92^; Aruta^51^; Lin and Wang^76^). For instance, Carrol Steward et al.^86^ emphasized that their study involved a single partner school, limiting its generalizability to other schools and contexts. Gutierrez et al.^53^ noted that their small sample of students in rural STEM-focused middle schools in the southeastern United States limits generalizability because not all students decided to participate. DeCamp^40^ noted that their exploratory study with a small faculty and student population does not aim to provide generalizable results, as it was intended to observe trends in their small sample. Thacker^77^ highlighted that their sample was predominantly female, suggesting that future studies should recruit more nationally representative samples.

Additionally, researchers emphasized the need for more reliable and validated measurement scales for assessing climate knowledge (e.g., Kolenatý et al.^7^; Lin and Wang^76^). Some researchers developed new climate knowledge scales specifically for their studies, which require further validation in future research.^72^^,^^73^ Additionally, Aruta^51^ relied on only two items as indicators of climate change knowledge. Fischer et al.^108^ also noted this limitation, indicating that their results may not generalize to all possible climate change statements. In conclusion, future research should focus on refining and developing a standardized, reliable, and validated instrument for climate knowledge, covering the most commonly measured dimensions (see Figure 4). That would lead to easier comparison of findings across studies and countries. For instance, a widely used climate knowledge measurement developed by Tobler et al.^17^ categorizes climate knowledge into four domains: physical knowledge about CO_2_ and the greenhouse effect, knowledge about climate change and causes, knowledge regarding expected consequences of climate change, and action-related knowledge.

Authors of the studies in our sample mentioned several future research directions, for example the need for comparison of climate knowledge across different age groups and countries.^72^^,^^73^ Other authors, such as Aruta^51^ and Dijkstra and Goedhart,^42^ emphasize that future studies should include a higher number of items/questions to ensure the reliability of participants’ responses. Additionally, some authors recommend the use of in-depth interviews to gain a broader understanding of respondents’ knowledge.^69^^,^^92^ This approach could enhance our understanding of differences in climate knowledge across different communities and across different demographic groups.^55^^,^^69^

Additionally, longitudinal research is needed to examine attitudes, behaviors, and knowledge, as well as the formation and persistence of the latter over time.^37^^,^^47^^,^^59^^,^^90^ There is also a need for greater education that can help translate knowledge into behavior.^109^ This education should focus on environmental science and climate change topics such as causes, mechanisms, and processes, which are not adequately covered by current curricula or are not well understood by students.^64^^,^^65^ Future studies should also examine how climate topics are taught in schools (e.g., which teaching methods are used and how effective they are).^88^ McNeill and Vaughn^13^ emphasize that future research should not only focus on scientific knowledge and science curriculum but also on individual and community-level actions to combat climate change. Additionally, Tranter,^31^ Nussbaum et al.^35^ and Acevedo et al.^74^ emphasize the need to examine whether increased knowledge of climate change leads to positive behavioral changes.

The reviewed studies demonstrate the need for methodological improvements, including the use of standardized, validated instruments and larger, representative samples. Additionally, there is a need for comparisons of climate knowledge across different age groups and countries, for longitudinal studies and in-depth interviews to better understand respondents’ climate knowledge. Lastly, future efforts should prioritize the integration of climate topics into education and explore their impact on behavior change.

## Discussion and conclusion

### Synthesis of key findings

To our knowledge, this systematic literature review is the first attempt to systematically analyze the content and dimensions measured in climate knowledge surveys. In the final sample of our review (*n* = 92), the majority of studies originated from North America (e.g., Bedford^38^; Gutierrez et al.^53^; Asgarizadeh et al.^58^; Carrol Steward et al.^86^), followed by Europe (e.g., Meira-Cartea et al.^103^; Harker-Schuch et al.^44^; Ratinen and Uusiautti^72^; Jurek et al; ^97^ Kurowski et al.^62^; Nepras et al.^18^).

The predominance of research conducted in North America and Europe makes it harder to draw meaningful comparisons and identify regional differences in climate knowledge across continents. For example, research indicates that different cultural contexts shape understanding of climate change.^110^^,^^111^ Future research should focus on the inclusion of underrepresented geographical contexts to provide a more comprehensive understanding of climate knowledge.

The primary researched population was the general public (e.g., Ebuehi and Olusanya;^101^ Fischer et al.^108^; Banwell et al.^96^; Alenda-Demoutiez^30^; Connor et al.^57^; Abunyewah et al.^49^; Adu-Boateng et al.^41^). The next most often researched population was primary and secondary school students (e.g., Dijkstra & Goedhart^42^; Bodzin et al.^14^; DeWaters et al.^9^; Flora et al.^34^; Meira-Cartea et al.^103^; García-Vinuesa et al.; ^67^; Aruta;^51^ Carrol Steward et al.^86^; Liarakou et al.^112^; and Karpudewan et al.^93^). A minority of studies examined climate knowledge among university students, pre-service teachers, and teachers (e.g., Borhan and Ismail^47^; Huxster et al.^64^; Boon^12^; Asshoff et al.^36^; Gazzaz and Aldeseet^65^; Nyarko and Petcovic^92^; Liu et al.^85^; Fernández et al.^37^; Jama et al.^75^). Future research should prioritize assessing the climate knowledge of teachers, as they are crucial for passing this knowledge to future generations.^12^^,^^37^^,^^46^^,^^47^

Our analysis revealed significant variation in how studies operationalized climate knowledge, with the number of items used for assessment ranging from 2 to 81. Gutierrez et al.^53^ used the most items, with 81 assessing climate knowledge (76 for objective and 5 for subjective knowledge). Additionally, 26 studies included assessment of subjective knowledge (e.g., McCright ^32^; DeWaters et al.^9^; Fischer & Said^91^; García-Vinuesa et al.^67^; Huxster et al.^64^) or self-reported knowledge.^89^ Objective knowledge of climate change, focusing on actual evidence or “factual knowledge”,^89^ was the most prevalent type of measure, consistent with previous research.^21^ Four studies also measured participants’ confidence in their objective knowledge.^56^^,^^80^^,^^91^^,^^92^ Stoutenborough and Vedlitz^113^ noted that subjective knowledge may not accurately capture scientific understanding compared to objective knowledge measures. Therefore, we recommend using both objective and subjective measures of climate knowledge, as relying solely on subjective knowledge is insufficient for a comprehensive understanding of climate change.

Studies included in our systematic literature review varied greatly in the dimensions of climate knowledge they incorporated. Most studies examined climate knowledge with three dimensions (e.g., Borhan and Ismail^47^; Anyanwu and Le Grange^46^; Fischer et al.^108^; Javeline et al.^99^; Gazzaz and Aldeseet^65^; Connor et al.^57^; Jurek et al.^97^; Abunyewah et al.^49^; Hurst Loo and Walker^59^; Yeh et al.^8^). Six studies used four dimensions (e.g., Meira-Cartea et al.^103^; Escoz Roldán et al.^78^; Harker-Schuch^44^), and three studies used five dimensions to measure climate knowledge.^21^^,^^76^ Our findings indicate there is a lack of consistency in how studies define and represent the different dimensions of climate knowledge. However, we find that the most commonly used dimensions for measuring climate change knowledge were general and physical knowledge, as well as the causes and consequences of climate change.

Lastly, some studies have emphasized that the validity of measuring climate knowledge is uncertain (e.g., Bodzin et al.^14^; Ratinen^73^). Therefore, as noted in several studies in our sample (e.g., Bodzin et al.^14^; Kolenatý et al.^7^; Lin and Wang^76^), future research should focus on refining and developing a standardized, reliable, valid, and comprehensive instrument for measuring climate knowledge. This will facilitate easier comparison of findings across studies and countries.

In summary, many studies have operationalized climate knowledge. Nevertheless, more studies focusing on regions outside of North America and Europe are needed. Additionally, there is a need for large-scale, cross-cultural, and longitudinal studies. Furthermore, the lack of standardized and validated measurement tools remains a challenge, limiting comparability across studies. Future research should focus on developing comprehensive instruments that measure both objective and subjective dimensions of climate knowledge. Additionally, such an instrument should include the most widely implemented dimensions for climate knowledge operalization in our sample (general and physical knowledge, and knowledge of the causes and consequences of climate change). Finally, more research that focuses on climate knowledge of teachers is needed as they play a key role in educating younger generations.

Our findings are valuable for researchers as a tool to evaluate climate knowledge, offering a synthesis of key insights from 92 different articles. They provide information about measurement instruments and future research implications, helping to comprehensively assess climate change knowledge among students, teachers and general population. Additionally, we synthesize the limitations and future directions proposed by the studies in our sample. Lastly, our findings can help policymakers and educators assess different measuring practices of climate knowledge. This can aid in implementing important changes to school curricula and educational policies to enhance climate change knowledge.

### Limitations of the study

Our systematic literature review has several limitations. First, despite employing a broad keyword search encompassing terms like “environmental literacy,” “environmental knowledge,” and “climate literacy,” it is possible that relevant studies were overlooked. Moreover, the strict inclusion and exclusion criteria might have inadvertently excluded valuable research of partial relevance to our review. For example, limiting the time frame allowed us to focus on studies most relevant to contemporary challenges and developments in the field.^114^^,^^115^ However, this approach may have excluded earlier work on climate knowledge. Finally, although we did not include gray literature, the screening process may still be susceptible to selection bias. Studies that did not align perfectly with our inclusion criteria may have been excluded, affecting the overall assessment of climate knowledge measurements.

## Acknowledgments

The study was conducted within research project V5-2374, “Climate Literacy and Environmental Behavior Among Young People in Slovenia and Europe: An Analysis of the Current State, Determinants, Consequences and a Proposal of Solutions,” funded by the Slovenian Research and Innovation Agency and Ministry of the Environment, Climate and Energy.

During the preparation of this work the author Ž.P. used ChatGPT in order to improve the readability and understandability of several sentences of the text. After using this tool, the authors reviewed and edited the content as needed and take full responsibility for the content of the publication.

## Author contributions

Ž.P. led the study selection proces; Ž.P. and M.L. conducted the analyses in the systematic review, and co-wrote the original draft of the manuscript. A.K. supervised the study, provided critical guidance on the research design and methodology, and contributed to the interpretation of findings. All authors are accountable for all aspects of the final systematic literature review.

## Declaration of interests

The authors declare no competing interests.

## Supplemental information

Table S1. Quality assessment of included studies

Table S2. Main Characteristics

Table S3. Subjective and objective knowledge

List S4. List of studies in the sample
