# Supplementary material for: Measuring climate knowledge: A systematic review of quantitative studies
Source: iScience. 2025 Jan 25;28(2):111888. doi: 10.1016/j.isci.2025.111888 (PMC11869530; doi:10.1016/j.isci.2025.111888)
Supplement: Table S1. Quality assessment of included studies [file mmc1.pdf]

iScience, Volume *28*

Supplemental information

Measuring climate knowledge: A systematic review of quantitative studies

Maruša Lubej, Žiga Petraš, and Andrej Kirbiš

Author & Year	Standard 1: Provides clear argument that links theory and research and demonstrates coherent chain of reasoning. Explicates theoretical and previous research in a way that builds the formulation of the question(s).		Standard 2: Applies rigorous, systematic, and objective methodology to obtain reliable and valid knowledge relevant to educational activities and programs.				Standard 3: Presents finding(s) and makes claims that are appropriate to and supported by the methods that have been employed.	Assessed quality
	1.1 Explicates theory and/or previous research that builds the formulation of the study aims.	1.2 Explicitly links findings to previous theory and research or argument for study.	2.1 Ensures that methods are presented in sufficient detail and clarity to clearly visualize procedures.	2.2 Provides evidence of reliability (collected data, information about instrument development, study populations).	2.3 Provides evidence of validity (collected data, information about instrument development).	2.4 Describes different characteristics of participants.	3.1 Findings and conclusions are legitimate and consistent with data collected.	
Abunyewah et al., 2023	1	1	1	1	1	1	1	7
Adu-Boateng et al., 2023	1	1	0	0	0	1	1	4
Alenda-Demoutiez, 2022	1	1	1	1	0	1	1	6
Anyanwu and Le Grange, 2017	1	1	1	1	0	1	1	6
Aruta, 2023	1	1	1	1	1	0	1	6
Asgarizadeh et al., 2023	1	1	1	1	1	1	1	7
Asshoff et al., 2021	1	1	1	1	1	1	1	7
Banwell et al., 2020	1	1	0	1	1	1	1	6
Bedford, 2016	1	1	1	1	0	1	1	6
Bodzin et al., 2014	1	1	1	1	1	1	1	7
Boon, 2016	1	1	0	1	1	1	1	6

Borhan & Ismail, 2011	1	1	1	1	0	0	1	5
Bozoglu et al., 2022	1	1	1	1	1	0	1	6
Bremer & Linnenluecke, 2017	1	1	1	1	1	1	1	7
Carroll Steward et al. , 2023	1	1	1	0	1	1	1	6
Chuvieco et al., 2021	1	1	1	0	0	0	1	4
Connor et al., 2022	1	1	1	1	0	1	1	6
Das et al., 2022	1	1	1	1	1	0	1	6
DeCamp, 2024	1	1	1	0	0	0	1	4
DeWaters et al., 2014	1	1	1	1	1	1	1	7
Di Gusto et al., 2018	1	1	1	1	1	1	1	7
Dijkstra & Goedhart, 2012	1	1	1	1	1	1	1	7
Ebuehi & Olusanya, 2013	1	1	1	0	1	1	1	6
Escoz Roldán et al., 2019	1	1	1	1	0	1	1	6
Fernández et al., 2023	1	1	1	1	1	1	1	7
Fischer et al., 2019	1	1	1	1	0	1	1	6
Fischer & Said, 2021	1	1	1	0	0	1	1	5
Flora et al., 2014	1	1	1	1	1	1	1	7

García-Vinuesa et al., 2021	1	1	1	1	1	1	1	7
Gazzaz & Aldeseet, 2021	1	1	1	1	1	1	1	7
Geiger et al., 2014	1	1	1	1	1	1	1	7
Gutierrez et al., 2022	1	1	1	1	1	1	1	7
Hallar et al., 2011	1	1	1	0	0	0	1	4
Harker-Schuch, 2020	1	1	1	0	0	1	1	5
Helbling et al., 2021	1	1	1	0	0	1	1	5
Higuchi et al., 2018	1	1	1	1	1	1	1	7
Hu et al., 2017	1	1	1	0	1	1	1	6
Hurst Loo & Walker, 2023	1	1	1	1	1	1	1	7
Huxster et al., 2015	1	1	1	1	1	1	1	7
Jama et al., 2023	1	1	1	1	1	1	1	7
Javeline et al., 2019	1	1	0	0	0	1	1	4
Jurek et al., 2022	1	1	1	1	0	1	1	6
Karpudewan et al., 2014	1	1	1	1	1	1	1	7
Karpudewan & Mohd Ali Khan, 2017	1	1	1	1	1	1	1	7
Klappa & Bouvier-Brown, 2021	1	1	1	0	1	1	1	6
Kolenatý et al., 2022	1	1	1	1	1	1	1	7

Kumar et al., 2023	1	1	1	0	0	1	1	5
Kurowski et al., 2022	1	1	1	1	1	1	1	7
Liarakou et al., 2010	1	1	1	1	1	1	1	7
Lin & Wang, 2023	1	1	1	1	1	0	1	6
Liu et al., 2022	1	1	1	1	1	1	1	7
Liu et al., 2014	1	1	1	0	0	0	1	4
McCright, 2010	1	1	1	1	0	1	1	6
McNeill & Vughn, 2012	1	1	1	0	1	1	1	6
Meira-Cartea et al., 2018	1	1	1	1	1	1	1	7
Mumpower et al., 2016	1	1	1	0	0	1	1	5
Nepras et al., 2023	1	1	1	1	1	1	1	7
Ngo et al., 2020	1	1	1	0	1	1	1	6
Nussbaum et al., 2015	1	1	1	1	1	1	1	7
Nyarko & Petcovic, 2021	1	1	1	1	1	1	1	7
Pan et al., 2023	1	1	1	1	0	1	1	6
Peterson & Kozlowski, 2024	1	1	1	1	1	1	1	7
Player et al., 2023	1	1	1	1	1	1	1	7
Powers et al., 2021	1	1	1	1	0	1	1	6
Rahman et al., 2020	1	1	1	1	1	1	1	7

Ratinen, 2021	1	1	1	1	1	1	1	7
Ratinen & Uusiautti, 2020	1	1	1	1	1	1	1	7
Regassa & Stoecker, 2014	1	1	1	1	1	1	1	7
Révolo Acevedo et al., 2022	1	1	1	1	1	0	1	6
Rooney-Varga et al., 2018	1	1	1	1	1	1	1	7
Rooney-Varga et al., 2021	1	1	1	1	1	1	1	7
Schollaert Uz et al., 2014	1	0	1	0	0	1	1	4
Seebauer, 2014	1	1	1	1	1	1	1	7
Siegner & Stapert, 2020	1	1	1	0	0	1	1	5
Sorensen et al., 2018	1	1	1	0	0	1	1	5
Stevenson et al., 2014	1	1	1	1	1	1	1	7
Stevenson et al., 2016	1	1	1	0	1	1	1	6
Taddicken et al., 2018	1	1	1	1	1	1	1	7
Thacker, 2023	1	1	1	1	0	1	1	6
Thaller & Brudermann, 2020	1	1	1	1	1	1	1	7
Tolppanen et al., 2023	1	1	1	1	1	1	1	7
Tranter, 2020	1	1	1	0	0	0	1	4
Tranter, 2021	1	1	1	0	0	0	1	4
Tranter et al., 2020	1	1	1	0	0	1	1	5

Trémolière & Djeriouat, 2021	1	1	1	0	0	1	1	5
Vainio & Paloniemi, 2013	1	1	1	1	1	1	1	7
Walker & McNeal, 2013	1	1	1	1	1	1	1	7
Wang et al., 2020	1	1	1	1	1	1	1	7
Wang et al., 2022	1	1	1	1	1	1	1	7
Woodika & Schoof, 2017	1	1	1	1	1	1	1	7
Yeh et al., 2024	1	1	1	1	1	1	1	7
Zhang et al., 2022	1	1	1	1	1	1	1	7
