# Supplementary material for: Measuring climate knowledge: A systematic review of quantitative studies
Source: iScience. 2025 Jan 25;28(2):111888. doi: 10.1016/j.isci.2025.111888 (PMC11869530; doi:10.1016/j.isci.2025.111888)
Supplement: Table S2. Main Characteristics [file mmc2.pdf]

Author & Year	Location	Sample	Age(s)	Study design	Instrument name of CK; number of items	Dimensions of CK	QA
Abunyewah et al., 2023	Ghana, Africa	n = 874 farmers	aged 18 to above 63 years old	Cross-sectional<br><br>empirical	Climate change knowledge; 16 items	Three kinds of knowledge concerning climate change:<br>1) Causes of climate change<br>2) Physical characteristics of climate change<br>3) Consequences of climate change	7
Adu-Boateng et al., 2023	Ghana. Africa	n = 367	Aged 20 to 60 years old	Cross-sectional, case-study	Household survey, 5 items		4
Alenda-Demoutiez, 2022	Africa (34 countries)	n = 45823 interviews	adults	cross-sectional	Afrobarometer survey; 3 items	Knowledge of climate change:<br>1) Causes<br>2) Consequences<br>3) Solutions	6
Anyanwu and Le Grange, 2017	Western Cape Province, Africa	n = 194	high school Geography teachers, below 40 years old (n = 39) and above 40 years old (n = 155)	cross-sectional	Climate change science literacy; 15 items	Knowledge component of climate change science literacy:<br>1) Climate processes and probable causes of climate change<br>2) Climate change impacts,<br>3) Climate change responses	6
Aruta, 2023	Philippines	n = 7233	15-year-old high school students from 187 schools across 17 regions in the Philippines	Cross-sectional	Climate change knowledge efficacy; 2 items		6
Asgarizadeh et al., 2023	USA, Canada	n = 323	aged 19 to 87 years old	cross-sectional	Climate change knowledge; 13 items	1) Physical knowledge<br>2) Causes<br>3) Consequences<br>4) Action-related knowledge	7
Asshoff et al., 2021	Germany	n = 110 students	pre-service biology teachers from the University of Muenster and Freiburg University of Education, mean age 23.76 years	experimental study, pretest/posttest	Climate change knowledge; 23 items	1) Stomata<br>2) Cycles and Global Change (comparison of preindustrial vs. present context with special focus on the carbon cycle)<br>3) Global Change Connections	7
Banwell et al., 2020	Chile	n = 454	aged 19 to 94 years old	mixed-methods approach	Climate change knowledge; 8 questions (number of items is unclear)		6

Bedford, 2016	USA	n = 458	university students	case study	Climate literacy scale; 15 items		6
Bodzin et al., 2014	USA	n = 868	urban eighth grade students', aged 13 to 15, four middle schools	Cross-sectional	Climate change knowledge; 28 multiple-choice items and three open-ended response items		7
Boon, 2016	Australia	n = 87	pre-service teachers, aged 17 to above 26 years old	longitudinal	Climate change knowledge; 7 multiple-choice items		6
Borhan & Ismail, 2011	Malaysia	n = 173	pre-service teachers enrolled in a Chemistry Teaching Methods course, third year of the teacher-education programme	cross-sectional	Climate change knowledge/environmental knowledge; 52 items	1) Causes<br>2) Consequences<br>3) Cures/solutions	5
Bozoglu et al., 2022	Turkey	n = 506	university students in 16 agricultural economics departments in Turkey	cross-sectional	Climate Change knowledge; 40 items		6
Bremer & Linnenluecke, 2017	Australia	n = 101	aged 18 to above 55, managers in the Australian energy industry	cross-sectional	Climate change knowledge; 17 items		7
Carroll Steward et al., 2023	USA	n (teachers) = 3<br>n (students) = 240	tenth grade (n = 4), eleventh grade (n = 210) and twelfth grade students (n = 26)	experimental study, pretest/posttest	Knowledge of climate change; 27 items	Knowledge of climate change (conceptual understanding of the Earth's climate and Global Climate Change)	6
Chuvieco et al., 2021	Spain	n = 845	aged 16 to more than 65 years old	Cross-sectional	Knowledge; 2 items		4
Connor et al., 2022	Vietnam	n = 410	aged 19 to 71 years old	cross-sectional	Knowledge about climate change; 8 items	1) Knowledge about CO2 and greenhouse effect'<br>2) Knowledge about the consequences of climate change'<br>3) Additional question by the authors of the study	6
Das et al., 2022	India	n = 200	farmers, 100 each in coastal and non-coastal NICRA districts	cross-sectional	Climate knowledge; 10 items	1) Climate change causes<br>2) Climate change consequences	6
DeCamp, 2024	USA	n = 149	university students of introduction Engineering, introductory Ecology, advanced Ecology, anthropology of climate change and advanced Family Policy	case study	Climate Literacy Survey; 76 quantitative items (35 questions)	Knowledge:<br>1) Climate Science/Mitigation Section<br>2) Climate Change Adaptation<br>3) Climate Justice	4

DeWaters et al., 2014	USA	n (middle school students) = 227<br>n (high school students) = 200<br>n (teachers) = 12	teachers from middle and high school and middle school and high school students	experimental	Climate Literacy Survey Instrument; 21 items (Middle school) + 1 self-assessment item; 23 items (High school) + 1 self-assessment item	Cognitive domain:<br>1) Climate science<br>2) Causes and impacts of climate change<br>3) Mitigating climate change<br>4) General knowledge<br>5) Self-assessment	7
Di Gusto et al., 2018	Taiwan	n = 1118	university students at nine universities across Taiwan, mean age = 21.4	cross-sectional	Climate change knowledge; 15 multiple-choice items		7
Dijkstra & Goedhart, 2012	five EU countries (France, Norway, Italy, The Netherlands and Spain)	n = 671	aged 12 to 21 years old, secondary school students participated in a CarboSchools project	Development and validation	Knowledge test; 12 items		7
Ebuehi & Olusanya, 2013	Nigeria, Africa	n = 410	adults, residents in Ifo LGA, Ogun State, South West, Nigeria	cross-sectional	Knowledge on climate change; 14 items with 6 being multiple-choice items		6
Escoz Roldán et al., 2019	Spain	n = 398	university students, aged 18 to 25 years old	mixed methods	Knowledge of climate change; 35 items	1) Causes of climate change<br>2) Consequences of climate change<br>3) Biophysisal processes of climate change<br>4) Responses and/or solutions to climate change	6
Fernández et al., 2023	Spain	n = 102	university students, pre-service primary teachers	using a pre- and post-test design	Knowledge-content questionnaire (KCCQ); 36 items	1) Climate and climate change science<br>2) Greenhouse gases and greenhouse effect<br>3) Causes and consequences of climate change	7
Fischer et al., 2019	Germany	n = 509	German citizens, the sample was nationally balanced in terms of gender, age and geographical distribution	cross-sectional	Climate change knowledge; 8 items (7 were in the analysis)	Knowledge domains:<br>1) State<br>2) Causes of climate change<br>3) Consequences of climate change	6

Fischer & Said, 2021	Germany	Study 1<br>n = 509	German citizens, aged 18 to 88 years old	cross-sectional	Climate change knowledge; 8 items		5
Flora et al., 2014	USA	n = 779 (that received the intervention)<br>n = 1241 (panel)	high school students, ninth to twelfth grade	pre- and post-assembly	Climate science knowledge; 10 items		7
García-Vinuesa et al., 2021	Portugal	n = 219	aged 15 to 18 years old, three schools located in the north of Portugal	cross-sectional	Climate change knowledge; 32 items	Climate change knowledge	7
Gazzaz & Aldeseet, 2021	Jordan	n = 285	university students from public schools, undergraduate	cross-sectional	Knowledge of Climate Change; 30 items	Knowledge of:<br>1) The nature of climate change<br>2) Causes of climate change<br>3) Effects of climate change	7
Geiger et al., 2014	Argentina, Colombia, Latin America	n (Argentina) = 168<br>n (Colombia) = 130	Argentina, aged 17 to 80 years old; Colombia, aged 18 to 53 years old	cross-sectional	Environmental knowledge; 36 items (12 items for each knowledge type)	1) System knowledge<br>2) Action related knowledge<br>3) Efficiency knowledge	7
Gutierrez et al., 2022	USA	n (quantitative data) = 97<br>n (qualitative data) = 113	aged 11 to 14 years old, middle school students	cross-sectional	Objective knowledge: climate literacy; 76 items		7
Hallar et al., 2011	USA	n (prequiz) = 214 students<br>n (postquiz) = 228 students	fifth and sixth grade students	experimental	Climate change knowledge; 6 items		4
Harker-Schuch, 2020	Austria and Australia	n = 401 students	aged 12 to 13 years old	experimental	Climate literacy questionnaire; 19 items	Knowledge domains:<br>1) Earth in the Solar System<br>2) GHGs as molecules<br>3) Albedo<br>4) Earth's Atmosphere	5
Helbling et al., 2021	Africa (30 countries)	n = 37,000	adults from 30 African countries	cross-sectional	Afrobarometer survey; 3 items	Knowledge about climate change and its long-term consequences	5
Higuchi et al., 2018	Brazil	n = 400	students from public and private education institutions in Manaus, Amazonas, 200 high school students, with a mean age of 16.70, the remaining 200	cross-sectional	Knowledge; 15 items		7

			were university students, with a mean age of 25.01				
Hu et al., 2017	USA	n = 464	the average age was 37.51	cross-sectional	Climate change knowledge; 8 items		6
Hurst Loo & Walker, 2023	USA	n = 205	aged 18 to 75 years old	cross-sectional	Climate change knowledge; 12 items	1) Climate change causes knowledge<br>2) Climate change physical knowledge<br>3) Climate change consequences knowledge.	7
Huxster et al., 2015	USA	n = 465	a sample of junior and senior students at two public, central East Coast universities	cross-sectional	Knowledge variables; 35 items for the calculation of Knowledge Score		7
Jama et al., 2023	Somalia, Africa	n = 434	students from three universities, Somali land (n=157) Puntland (n=145) and from South Central Somalia (n=132)	cross-sectional	Climate Change Knowledge (CCK); 4 items		7
Javeline et al., 2019	USA	n = 662	North Carolinians, homeowners	cross-sectional	Knowledge; 3 questions (number of items is unclear)	1) Knowledge of climate change causes<br>2) Knowledge of climate-related hazards<br>3) Knowledge of thermal expansion	4
Jurek et al., 2022	Czech Republic	n (primary school students) = 758<br>n (secondary school students) = 462	22 primary and 17 secondary schools	cross-sectional	Knowledge of climate change; 6 open-ended questions (number of items is unclear)	1) Causes and consequences of climate change<br>2) Factual knowledge of the greenhouse gases<br>3) Principle and underlying logic of greenhouse effect	6
Karpudewan et al., 2014	Malaysia	n = 73<br>(35 in experimental and 38 in control group)	Secondary school students	Experimental	AREPDiT (atmosphere-related environmental problem diagnostic test; 13 items		7
Karpudewan & Mohd Ali Khan, 2017	Malaysia	N = 62, 30 in experimental group and 32 in control group	62 sixteen year old students	experimental	CCKT, 5 items		7
Klappa & Bouvier-Brown, 2021	USA	n (First-year control) = 74<br>n (First-year exposure) = 37	first year students and non-first year students	experimental	Survey; 12 questions (number of items is unclear)	Global Warming/Climate Change (focused on students' knowledge of global warming)	6

		n (Non-first-year interested with exposure) = 38					
Kolenatý et al., 2022	Czech Republic	n (pre-test) = 429<br>n (post-test) = 188<br>n (final sample) = 123	aged 12 to 17 years old, seventh–ninth graders from 47 schools	cross-sectional, quasi-experimental	Climate change knowledge; 15 items	1) System knowledge<br>2) Action knowledge,<br>3) Effectiveness knowledge,	7
Kumar et al., 2023	UK	n = 71	aged 9 to 14 years old, fifth to ninth grades	experimental	Quiz for primary school students with 10 multiple-choice items; quiz for secondary school students with a mix of 14 multiple-choice items with 6 descriptive open-ended items (with a word limit of 50)	Knowledge of climate change:<br>1) Causes,<br>2) Impact,<br>3) Mitigation	5
Kurowski et al., 2022	Poland	n = 1000	aged 18 to above 64 years old	cross-sectional	Knowledge and self-assessment about knowledge of global warming and climate change; 4 questions (number of items is unclear)	1) Knowledge about the cause of global warming<br>2) Knowledge about the mechanism of global warming<br>3) Knowledge about the effects of global warming<br>4) Subjective knowledge	7
Liarakou et al., 2010	Greece	n = 626	Secondary school students (8th to 11th grade)	Cross-sectional	Greenhouse effect, 22 items	1) Causes<br>2) Impacts<br>3) Solutions	7
Lin & Wang, 2023	Taiwan	Study 1<br>n = 510 valid questionnaires<br>Study 2<br>n = 397 valid questionnaires	**Study 1:**<br>slightly more respondents were male (51.6%) and most were aged between 21 and 30 or 31 –40 (32.3% and 32.1%, respectively)<br>**Study 2:**<br>approximately 64.6% of respondents were men, who were predominantly aged between 41 and 50 years (47%	cross-sectional	Study 1<br>Climate change knowledge; 37 items<br>Study 2<br>Climate change knowledge; 12 items	Study 1<br>5 dimensions:<br>1) Causal knowledge<br>2) Basic knowledge<br>3) Effect knowledge<br>4) Action-related knowledge<br>5) Procedural knowledge<br>Study 2<br>4 dimensions:<br>1) Causal knowledge<br>2) Basic knowledge<br>4) Action-related knowledge<br>5) Procedural knowledge	6

Liu et al., 2022	USA	enrolled in the course<br>n = 184 PSTs<br>matched data set<br>n = 100	pre-service science teachers, university students	cross-sectional	Climate change knowledge; 11 items		7
Liu et al., 2014	USA	n = 481	aged 21 to 95 years old, farmers and ranchers from Nevada	cross-sectional	Climate change knowledge; 2 items		4
McCright, 2010	USA	2001 poll: n = 1060<br>2002 poll: n = 1006<br>2003 poll: n = 1003<br>2004 poll: n = 1005<br>2005 poll: n = 1004<br>2006 poll: n = 1000<br>2007 poll: n = 1009<br>2008 poll: n = 1012	Adults	cross-sectional	Climate change knowledge; 3 items		6
McNeill & Vughn, 2012	USA	n = 75	aged between 16 and 18 years old, eleventh and twelfth grade students from three different high schools in the same large urban school district in the USA	cross-sectional	Conceptual understanding of climate change; 6 multiple-choice items + 1 open-ended item		6
Meira-Cartea et al., 2018	Spain	n = 1149	university students from 15 undergraduate degree courses	cross-sectional	Knowledge of climate change; 32 Likert-type items	Students' knowledge of CC:<br>1) Causes of CC<br>2) Consequences of CC<br>3) Biophysical processes of CC<br>4) Actions, responses and solutions to CC	7
Mumpower et al., 2016	USA	n = 1321	adults, data from a US national public opinion survey, the sample was drawn from KnowlegePanel, a probability-	cross-sectional	Climate Change Knowledge (CCK); 10 items		5

			based web panel designed to be representative of the USA for adults aged 18 and over				
Nepras et al., 2023	Three EU countries (Czech Republic, UK, Portugal)	n (total)= 473<br>Czech Republic = 304<br>UK = 125<br>Portugal = 44	primary school students	cross-country	Climate change knowledge; 8 items		7
Ngo et al., 2020	Vietnam	n = 1086	aged 16 to 90 years old, with 49% of the sample 50 years and older, from Quy Nhon, Can Tho, and Da Nang	cross-sectional	Individual knowledge; 3 questions (2 multiple-choice) (number of items is unclear)		6
Nussbaum et al., 2015	USA	n = 119	randomly assigned seventh graders (middle school) to either a game-based condition or control condition, middle school	experimental<br><br>pretest/posttest (and delayed posttest)	Content knowledge; 22 items<br><br>knowledge about the greenhouse effect and climate change		7
Nyarko & Petcovic, 2021	Ghana, Africa	Pilot study:<br>n = 11<br>Final study population:<br>n = 225	**Pilot study:**<br>Ghanian students, 18-30 years old, studying educational programmes in USA universities<br>**Final study sample:**<br>18-20 years old, first and secondary year elementary preservice science teachers recruited from three colleges of education in Ghana, dominantly male	cross-sectional<br><br>mixed method, descriptive study	Knowledge about ozone depletion and knowledge about climate change;<br>42 items		7
Pan et al., 2023	China	n = 3067	aged under 17 to above 65 (80.3% are between 18 and 34) years old	cross-sectional	Climate change literacy; 30 items	1) Causes of climate change<br>2) Consequences of climate change<br>3) Human engagement (policy issues)<br>4) Physical (general) knowledge	6
Peterson & Kozlowski, 2024	USA	(pilot) study 1: n = 155<br>study 2: n = 714<br>study 3: n = 684	**Study 1:**<br>Participants in this sample ranged in age from 18 to over 74<br>**Study 2:**<br>Participants in this sample	Development and validation	Climate Change Counseling Scale (3CS); 7 items		7

			ranged in age from 18 to over 74				
Player et al., 2023	UK	n = 346	undergraduate students, mean age = 19.06 years	validation	Environmental knowledge; 19 items	1) System knowledge<br>2) Action-related knowledge<br>3) Effectiveness knowledge	7
Powers et al., 2021	USA	n = 89	undergraduate students, primarily juniors (30 %) and seniors (61 %) from a range of engineering disciplines	experimental	Climate Literacy Questionnaire; 43 items	1) Cognitive scale<br>2) a self-assessment of their knowledge about global CC	6
Rahman et al., 2020	Bangladesh	n = 1500	university students, across 16 public universities located in 14 distinct districts in Bangladesh	cross-sectional	Climate change and dengue fewer knowledge; 2 questions		7
Ratinen, 2021	Finland	n = 950	elementary and secondary students, fifth-ninth graders, the average age was 13.6 (10% were fifth-graders, 20% sixth-graders, 15% seventh-graders, 31% were eighth-graders, and 24% were ninth-graders)	cross-sectional	Climate change knowledge; 13 items		7
Ratinen & Uusiautti, 2020	Finland	n = 950	elementary and secondary students, fifth-ninth graders, the average age was 13.6 (10% were fifth-graders, 20% sixth-graders, 15% seventh-graders, 31% were eighth-graders, and 24% were ninth-graders)	cross-sectional	Climate change knowledge; 9 item		7

Regassa & Stoecker, 2014	Ethiopia, Africa	n = 2188	1,094 men and 1,094 women	cross-sectional	Climate change knowledge; 11 items		7
Révolo Acevedo et al., 2022	Peru, South America	n = 382	inhabitants of the the Chilca district, aged between 20 and 50 years old	cross-sectional	Environmental knowledge; 23 items	1) Questions about air and soil<br>2) Questions of biodiversity<br>3) Questions about climate change	6
Rooney-Varga et al., 2018	North and South America, Europe and Africa	n = 2,042 participants	aged 11 to more than 75, people from eight nations	cross-country<br><br>pre-/post-survey	Climate change - knowledge;<br>8 questions subjective (1 question) & objective knowledge	1) Knowledge about climate change causes<br>2) Knowledge about climate change impacts	7
Rooney-Varga et al., 2021	USA	n = 2,080 participants	aged 14-75 years old	cross-sectional<br><br>pre-/post-survey	Climate change - knowledge;<br>6 items	1) Knowledge about climate change impacts	7
Schollaert Uz et al., 2014	USA	n = 72 sixth graders<br>n = 64 fifth graders<br>n = 10 teachers<br>n = 15 teachers	fifth and sixth graders and teachers, divided into:<br>1) activity & live SOS show: n= 44<br>2) activity & information, NO SOS: n = 38<br>3) live SOS show: n = 28<br>4) autorun SOS show: n = 43<br>5) control group, NO SOS: n = 8	experiment	Q&A: El Nin˜ o's Effect on Fish - Assessments; 8 multiple choice items and 2 open-ended items		4
Seebauer, 2014	Austria, USA, UK, Canada	n = 193	aged below 26 to above 35	validation	Climate change knowledge; 22 questions (30 items)		7
Siegner & Stapert, 2020	USA	n = 116 students	fifth and seventh grade students (control groups), eighth grade students	experimental, case study	Climate literacy - student survey; 17 items		5
Sorensen et al., 2018	USA	n (total) = 55<br><br>Site 1: n = 26<br>Site 2: n = 29	aged 18 to above 66 years old	experiment	Environmental identity and knowledge; 13 items		5

Stevenson et al., 2014	USA	n = 387	middle school students, aged 11 to 15 years old, sixth to eight graders	cross-sectional	Climate change knowledge; 17 items	1) Climate change science<br>2) Climate change causes<br>3) Climate change impacts	7
Stevenson et al., 2016	USA	n = 369	middle schol students, aged 11 to 15 years old, sixth to eight graders	cross-sectional	Climate change knowledge; 19 items	1) Climate change science<br>2) Climate change causes<br>3) Climate change impacts	6
Taddicken et al., 2018	Germany	n = 935	aged 14 to above 60 years old	longitudinal study, in three waves (from 2013 to 2014)	Knowledge about climate change; 37 items	1) Causal knowledge<br>2) Basic knowledge<br>3) Effects knowledge<br>4) Action-related knowledge<br>5) Procedural knowledge	7
Thacker, 2023	USA	total n = 605<br><br>There were three experimental conditions:<br>1) the estimation game intervention group (n = 204)<br>2) modified intervention group (n = 198)<br>3) control group (n = 203)	university students (undergraduate)	experimental	Climate change knowledge; 7 items		6
Thaller & Brudermann, 2020	Austria	n = 499	The sample is representative with regard to gender, places of residence and age (the youngest age group was slightly underrepresented)	cros--sectional	Climate change knowledge; 10 items		7
Tolppanen et al., 2023	Finland	n = 1703	tenth to twelfth grade high school students	cross-sectional	Climate change knowledge; 10 items		7
Tranter, 2020	Australia	n = 1317 (AuSSA)	Data are from the 2017 Australian Survey of Social Attitudes (AuSSA), a national social survey designed to be representative of the Australian adult population.	cross-sectional	Climate change knowledge/Polar knowledge; 3 questions<br>self-assessed knowledge; 1 question		4

Tranter, 2021	Australia	n = 1287 (AuSSA)	Data from the 2018 Australian Survey of Social Attitudes (AuSSA) are analyzed. The AuSSA is a national survey first administered in 2003, with participants selected at random from the Australian Electoral Roll to be representative of the Australian adult population.	cross-sectional	Climate change knowledge/Polar knowledge and Raising Sea Levels; 3 questions<br>self-assessed knowledge; 1 question		4
Tranter et al., 2020	Australia	n = 1287 (AuSSA)<br>n = 1593 (Our Lives Wave 7; 2019)	The data analysed here are from two sources, the 2018 Australian Survey of Social Attitudes (AuSSA) and the 2019 Our Lives survey of young people from the state of Queensland (QLD), Australia.	cross-sectional	Climate change knowledge/Polar knowledge and Raising Sea Levels; 3 questions<br>self-assessed knowledge; 1 question		5
Trémolière & Djeriouat, 2021	USA	Study 1:<br>n = 479<br>Study 2:<br>n = 496<br>Study 3:<br>n = 397	**Study 1:**<br>American participants were recruited on the Amazon Mechanical Turk online data collection platform. Mean age = 37.6<br>**Study 2:**<br>American participants were recruited on the Amazon Mechanical Turk online data collection platform. Mean age = 40.2<br>**Study 3:**<br>American participants were recruited on the Amazon Mechanical Turk online data collection platform. Mean age = 37.6	cross-sectional	Climate change knowledge; 19 items	1) General and causal knowledge dimension<br>2) Physical knowledge dimensions<br>3) Consequence knowledge dimensions	5

Vainio & Paloniemi, 2013	Finland	n = 1004	citizens who were 15 years or older	cross-sectional	Climate change knowledge; 3 items		7
Walker & McNeal, 2013	USA	n = 122	students and teachers, 62% reported their occupation as educator, 16% were students, and the remaining 22% represented administrators, scientists, unemployed, and other	Development and validation	The Knowledge Dimension; 27 items	The Knowledge Dimension scales/sub-scales were:<br>(1) Impacts of Climate Change<br>(2) Causes of Climate Change --> sub-scales of:<br>(2.1) Temperature<br>(2.2) Contribution<br>(3) Misunderstandings about Climate Change/sub-scales of:<br>(3.1) Misinformation	7
Wang et al., 2020	USA	n = 258	secondary agriculture teachers from fourteen states	cross-sectional	Conceptual knowledge about global climate change (GCC); 6 items		7
Wang et al., 2022	China	n = 8322	primary school children, fromn 12 cities across China	cross-sectional	Knowledge; 7 items		7

Woodika & Schoof, 2017	USA	n = 264	university students (undergraduate and graduate students)	cross-sectional	Knowledge questions; 12 items		7
Yeh et al., 2024	Taiwan	n = 2275	public officials, the age of most respondents ranged between 20 and 65	cross-sectional	CCL assessment; 14 questions (number of items is unclear)	Cognitive:<br>1) Issue knowledge<br>2) Content knowledge<br>3) Strategic knowledge)	7
Zhang et al., 2022	China	n = 2964	junior high school students from Beijing	cross-sectional	Climate change knowledge; 4 items		7

Note: CK = climate literacy; QA = Quality Assessment
