# Supplementary material for: Measuring climate knowledge: A systematic review of quantitative studies
Source: iScience. 2025 Jan 25;28(2):111888. doi: 10.1016/j.isci.2025.111888 (PMC11869530; doi:10.1016/j.isci.2025.111888)
Supplement: Table S3. Subjective and objective knowledge [file mmc3.pdf]

Authors and year	Items of subjective knowledge	Response options
**Adu-Boateng et al., 2023**	In the Table below indicate your awareness and knowledge about the causes and consequences of the listed climatic events in your community.	For each listed climatic event: flooding, coastal erosion, excessive heat and other, respondents had to indicate their awareness and knowledge about the causes and consequences.
**Aruta, 2023***	Explain how carbon-dioxide emissions affect global climate change.<br>Explain why some countries suffer more from global climate change than others.	4-point scale: 1 (I couldn't do this) to 4 (I could do this easily)
**Banwell et al., 2020**	How much do you know about climate change?	3-point scale: Nothing, A little bit, A lot
**DeWaters et al., 2014**	I feel I know (a lot/quite a bit) about global climate change.	5-part Likert-type scale with one neutral response: 1 (a lot) to 5 (quite a bit)
**Fischer & Said, 2021**	How certain are you that your answer is correct?	6-point scale: 50% (not at all certain, I was guessing) to 100% (certain, I know the answer)
**García-Vinuesa et al., 2021**	To what extent do you feel informed about different aspects of climate change?<br>About climate change in general.<br>About the causes of climate change.<br>About the measures against climate change.<br>About the consequences of climate change.	4-point scale: 1 (not informed) to 4 (well informed)
**Gutierrez et al., 2022**	Personally, how well informed do you feel about...<br>How the Earth's "climate system" works.<br>The different causes of global warming.<br>The different consequences of global warming.<br>Ways in which we can reduce global warming.	Student responses were coded as not at all (1), not very well (2), well (3), and very well (4)
**Huxster et al., 2015**	I know that changes in the atmosphere cause climate change, but I don't know very much about the scientific processes that make it happen.<br>No one has ever really explained to me how the greenhouse effect works, or if they have I can't really remember.	5-point Likert scale: 1 (disagree) to 5 (agree)
**Karpudewan et al., 2014**	Are you sure about your answer given to the previous two questions?	Yes/No
**Kurowski et al., 2022**	Please assess (on a scale from 0 - I have no knowledge, to 7 - I am very knowledgeable) your knowledge of global warming and climate change.	Assessment with one score from 0 (I have no knowledge) to 7 (I am very knowledgeable)

**McCright, 2010**	A straight forward measure of perceived understanding of global warming asked respondents to self-report how much they understand the issue of global warming.	4-point scale: 1 (not at all) to 4 (a great deal)
**Ngo et al., 2020**	Are you aware of causes of climate change)	Yes/No
**Nyarko & Petcovic, 2021**	Items assess both the accuracy of preservice teachers' knowledge and their confidence	4-point scale: 'I am sure this is wrong', 'I think this is wrong', 'I think this is right' and 'I am sure this is right'
**Powers et al., 2021**	How much do you feel you know about global climate change?	5-point scale; from A lot (A.) to Nothing (E.)
**Rooney-Varga et al, 2018, Rooney-Varga et al., 2021**	26 questions about urgency, hope, intent and values about climate change, for example:<br>How worried are you about climate change,<br>Feelings about climate change etc.	Not guilty to guilty, calm do outraged/angry, unconcerned to alarmed, not afraid to very afraid etc.
**Schollaert Uz et al., 2014**	1 open ended question:<br>Did you learn anything new from this video? If so, what?	Open-ended
**Seebaure, 2014**	Personally, how well informed do you feel you are about the different a causes of climate change?<br>Personally, how well informed do you feel you are about the different consequences of climate change?<br>Personally, how well informed do you feel you are about ways in which we can fight climate change?	4-point scale: Very well informed, Fairly well informed, Not very well informed, Not at all informed
**Siegner & Stapert, 2020**	Name one thing your community could realistically do to adapt or mitigate climate changes. How and why would your plan work?	Open-ended
**Thaller & Bruderman, 2020**	For each of objective knowledge questions, participants could choose one answer and additionally had to state the subjective probability that the answers were correct.	The options ranged from 50% (guessing) to 100% (fully confident)
**Tranter, 2020**	How much do you feel that you understand about climate change – would you say a great deal, a moderate amount, only a little, or nothing at all?	Scale ranging from 1 (nothing at all) to 4 (a great deal)
**Tranter, 2021**	How much do you feel that you understand about climate change – would you say a great deal, a moderate amount, only a little, or nothing at all?	Scale ranging from 1 (nothing at all) to 4 (a great deal)<br>Scale ranging from 0 (Not at all confident) to 100 (Totally confident)

	After answering each question, participants were instructed to indicate the extent to which they were confident that the response they just provided was the correct one.	
**Tranter et al., 2020**	How much do you feel that you understand about climate change – would you say a great deal, a moderate amount, only a little, or nothing at all?	Scale ranging from 1 (nothing at all) to 4 (a great deal)
**Trémolière & Djeriouat, 2021**	After answering each question, participants were instructed to indicate the extent to which they were confident that the response they just provided was the correct one.	Scale ranging from 0 (Not at all confident) to 100 (Totally confident)
**Vainio & Paloniemi, 2013***	Personally, do you think that you are well informed or not about…<br>… the different causes of climate change?<br>… the different consequences of climate change?<br>… ways in which we can fight climate change?	4-point scale: 1 (very well informed) to 4 (not informed at all)
**Zhang et al., 2022***	Has your school carried out poster design activities on energy conservation, emission reduction, low carbon and environmental protection?<br>Has your school organized a donation drive for used items?<br>Has your school provided the opportunity to participate in science fairs on carbon neutrality, low carbon environmental protection and other themes?<br>Has your school organized lectures in popularizing knowledge on carbon neutrality, energy conservation, emission reduction, low-carbon environmental protection and so on?	Responses were: 1 (yes), 2 (no; no idea)

**Authors and year**	**Items of objective knowledge**	**Response options**
**Abunyewah et al., 2023; Garcia-Vinuesa et al., 2021, Huxter et al., 2015; Jama et al., 2023; Kolenatý et al., 2022; Lin & Wang, 2023; Liu et al., 2014; Nepras et al., 2023; Ratinen, 2021;**	Adaption from Tobler et al, 2021 and Shi et al., 2016, 16 items such as:<br>Burning weeds, among other things, contribute to climate change.<br>The smoke released into the atmosphere during burning is a greenhouse gas.<br>Greenhouse gases are harmful to plant and animal growth.	5-point scale: 1(strongly disagree) to 5 (strongly agree);<br>in Garcia-Vinuesa et al., 2021 the scale was from 1 (totally agree) to 4 (totally disagree);<br>In Huxster et al., 2015 all items were measured with a 5-point agreement scale<br>In Jama et al., 2023 from 1 (strongly disagree) to 5 (strongly agree);<br>In Liu et al., 2014 the scale was from 1 (disagree) do 3 (agree);<br>In Nepras et al., 2023 the response options were I agree/I disagree/I don't know;<br>In Ratinen, 2021 and Ratinen & Uusiautii, 2020 scale was from 1 (strongly disagree) to 5 (strongly agree)

**Ratinen & Uusiautti, 2020; Regassa & Stoecker, 2014; Revolo Acevedo et al., 2022; Sorensen et al., 2018; Taddicken et al., 2018; Thacker, 2023; Woodika & Schoof, 2017; Liarakou et al., 2010**	The increase in greenhouse gas emissions is the main cause of climate change.<br>Human activities are a major cause of climate change.<br>Climate change is mainly due to natural causes.<br>Climate change will bring more negative than positive consequences to the world.<br>The number of hot days has increased over the past years.<br>Climate change's negative consequences limit farming outputs.<br>Similarly to the previous study Garcia-Vinuesa et al., 2021 had 32 items, for example:<br>The greenhouse effect is a natural phenomenon.<br>A warmer planet will expand the area of incidence of tropical diseases.<br>The increased temperatures will favour the concurence of extreme wheather events (cyclones, hurricanes, floods, etc.).<br>The polar hole in the ozone layer causes the melting of the poles.<br>If we stop emitting greenhouse gases, we will not be affected by climate change.<br>Skin cancers will increase as a result of climate change.<br>Acid rain is one of the causes of climate change.<br>Most of the greenhouse gases present in the atmosphere come from natural sources.<br>CO2 is the main gas responsible for climate change.<br>All countries will suffer climate change.<br>In Huxster et al., 2015, there were 35 items, such as:<br>We need to change our transportation system, like creating more public transportation, in order to make a big impact on reducing climate change<br>Individuals can reduce climate change by not buying gas-guzzling cars and instead maybe owning a more fuel-efficient car or hybrid	In Regassa & Stoecker, 2014 1 meant agree and 0 disagree;<br>In Revolo Acevedo et al., 2022 from 1(strongly agree) to 5(strongly disagree)<br>In Sorensen et al., 2018 from strongly disagree to strongly agree<br>In Taddicken et al., 2018 from 1 (disagree entirely) to 5 (agree entirely and in full)<br>In Thacker, 2023 from 1 (strongly disagree) to 5 (strongly agree);<br>In Woodika & Schoof, 2017 from 1 (strongly agree) to 4 (strongly disagree)<br>In Liarakou et al., 2010, 1- I agree, 2- I disagree, 3- I don't answer

The government could put more resources into developing alternative and renewable sources of energy in order to reduce climate change
I’ve heard that using efficient light bulbs, turning off electric appliances and insulating my house are all ways I can reduce my contribution to climate change
Even though it’s sometimes debated, I feel that it’s pretty well documented that humans have released too much carbon dioxide into the atmosphere
The government could tax carbon emissions or introduce a system of cap and trade in an effort to reduce climate change
The best way to deal with climate change would be to reduce or eliminate carbon-based fuel sources
Burning fossil fuels releases carbon dioxide into the atmosphere, which traps heat and causes climate change
Individuals can give political support to clean energy initiatives to help deal with climate change.
In Jama et al., 2023 there were 4 statements:
Forests regulate climate by capturing greenhouse gas emissions and increasing carbon removals from the atmosphere
Burning forests as charcoal have a potential consequence on climate change in Somalia
Strategies for forest management (i.e., forestation) can mitigate climate change
Increase in the climate change impacts is partly caused by deforestation
In Kolenaty et al., 2022 there were 43 questions with responses on the Likert scale and 2 open-response questions, for example:
Planet Earth is warming because… (tick all correct options):
... there are sunspots in the sun, the sun radiates more heat.

... the amount of greenhouse gases in the atmosphere is increasing.
... the earth 's core is heating up.
... the ozone layer functions as a greenhouse around the Earth.
... holes in the ozone layer let more heat from the sun go through.
... the ice age is ending.
... more thermal radiation reflected from the Earth remains in the atmosphere.
... the Earth's orbit around the Sun has changed.
Global warming / climate change has the following impacts (tick all that apply):
rising ocean levels
rising temperature (atmosphere, oceans, earth's surface)
frequent volcanic eruptions and earthquakes
melting ice (for example in the polar regions)
heating of the Earth’s core
less oxygen in the atmosphere
lack of water and food
more extreme weather (hurricanes, heat waves…)
skin cancer
enlarging holes in the ozone layer
In Lin & Wang, 2023 there were 57 questions included, such as:
Increased greenhouse gases are mainly caused by human activities.
The last century’s global increase in temperature was the largest during the past 1000 years.
The ‘90s were the warmest decade worldwide during the past century.
Today’s global CO_2 concentration in the atmosphere already occurred in the past 650,000 years.
Basic knowledge
Carbon dioxide (CO_2) is a greenhouse gas.
CO_2 is harmful to plants.

	The ozone hole is the main cause of the greenhouse effect.<br>Action-related knowledge<br>Reducing the temperature of a gas-heated room by 1◦ decreases CO2 emissions.<br>The production of 1 kg of beef produces more greenhouse gases than the production of 1 kg of wheat.<br>A large part of CO2 emissions in Taiwan is produced by power industries.<br>Procedural knowledge<br>Climate science findings are always afflicted with a sense of uncertainty.<br>Forecasts about climate change can only be assumed to be highly likely, they are never 100%.<br>In Liu et al., 2014 there were only 2 items:<br>I believe that we are in a period of climate change.<br>I believe that human activity has been playing a significant role in recent climate change.<br>In Nepras et al., 2023 there were 8 statements:<br>1. Taking a plane when going on vacation has a greater impact on the environment than riding a train.<br>2. Carbon dioxide generated by the combustion of coal in coal-fired power plants may affect the climate of our planet.<br>3. Nuclear power plants release more carbon dioxide into the atmosphere than coal-fired power plants.<br>4. The climate of our Earth has been warming in recent decades. One of the causes of this warming is human activity.<br>5. When a school class goes on a trip in six cars, it will have less impact on the environment than if the entire class takes one bus.<br>6. Cutting down tropical rainforests in Africa cannot have a significant impact on climate change in Europe.<br>7. The warming of the Earth's climate is causing ocean levels to rise as water from melting glaciers increases.<br>8. Wind and solar power plants generate large amounts of carbon dioxide.	

In Ratinen & Uusiautti, 2020 there were 17 items and in Ratinen, 2020, 31 items were used, such as: Climate change is because . . . ” and 13 items were used:
There is too much greenhouse gas
There is intensive forest logging
Nitrogen oxides are released from fertilizers
There is too much IR radiation that stays on the Earth
Purchased products generate greenhouse gases
Landfills produce methane
There is too much consumption of milk and dairy products
Emitted IR radiation is absorbed by greenhouse gases
We use fossil fuels such as oil and coal
the Earth has ozone zone
We are using nuclear power
We are using wind power
Factories and cars increase temperature etc.
In Regassa & Stoecker, 2014 there were 11 statements:
Effects of global warming have already begun.
Pollution from human activities is the primary cause of global warming.
Global warming threatens our way of life.
The effects of global warming are underestimated.
Overusing fertilizers and pesticides will damage the environment.
We are approaching the limit on the number of people the earth can support.
Humans’ interference with nature often produces disastrous consequences.
Humans are severely abusing the environment.
Plants and animals have as much right as people do to exist.
The earth is like a spaceship with very limited capacity and resources.
If things continue on their present course, we will soon experience a major ecological catastrophe.
In Revelo Acevedo et al., 2022, 23 items were included, such as:
Do you think there has been a lot of air pollution in your district in recent years?

	You believe that with the change in land use that responsible consumption has been taking place or has changed.<br>You, change agricultural practices implementing adapting to changes in the climate.<br>Do you think that you can be affected by a lot of air pollution in some activities that you develop.<br>You believe that on the importance of the territorial ordering of your locality.<br>You have attended a talk about air pollution and land use.<br>You believe in the last decades the level of agricultural production has dropped.<br>You believe that there is a decrease in livestock and pasture planting.<br>You consider that urbanization will bring effects and causes in changes in the soil environment.<br>He believes that the use of agrochemicals in soils affects biodiversity.<br>In Sorensen et al., 2018, 10 statements were included, such as:<br>If you notice trash/litter on the ground when you are outside in your community, how likely are you to pick it up and dispose of it in appropriate bin?<br>Preserving local biodiversity is important to me.<br>I would support policies to improve the Jamaica Bay ecosystem<br>I would be willing to pay up to $25 yearly to improve the Jamaica Bay ecosystem.<br>I consider myself an environmentalist.<br>I believe I can have an impact in solving environmental issues.<br>I am confused about what is good and what is bad for the environment.<br>I think climate change is caused by human actions/choices.<br>I think climate change will cause harm to people living in the Jamaica Bay in the future.<br>Global climate change is a very serious problem.<br>In Taddicken et al., 2018 37 statements were included, such as:	

1. The global CO_2 concentration in the atmosphere has increased during the past 250 years.
2. The increase of greenhouse gases is mainly caused by human activities.
3. With a high probability, the increase of CO_2 is the main cause of climate change.
4. Climate change is mainly caused by natural variations (such as changes in solar radiation intensity and volcanic eruptions).
5. The last century's global increase in temperature was the largest during the past 1,000 years.
6. The '90s were globally the warmest decade during the past century.
7. Today's global CO_2 concentration in the atmosphere already occurred in the past 650,000 years.

In Thacker, 2023 7 statements were included:

1.We cannot know about ancient climate change. (Reversed)
2.Earth's climate has probably changed little in the past. (Reversed)
3.Greenhouse gas levels are increasing in the atmosphere.
4.Earth's average temperature has increased over the past 100 years. This is evidence of climate change.
5.Average sea level is increasing. This is evidence of climate change.
6.Most of the world's glaciers are decreasing in size. This is evidence of climate change.
7.Most countries are committed to climate action.

In Woodika & Schoof, 2017 the 12 items were included:

Atmospheric scientists agree that humans are responsible for a substantial proportion of the global warming that has occurred in the last years.
Changes in the land surface contribute to climate change.

	A sizeable portion of the “ozone hole” resides over the United States.<br>The sun is the primary source of energy for earth’s climate system.<br>The earth’s average air temperature cannot change unless the amount of energy arriving from the sun changes.<br>Prior to the Industrial Revolution, the last years have been a relatively stable period in earth’s climate history.<br>Life impacts the earth’s climate and climate impacts life on Earth.<br>Climate variations are both natural and anthropogenic (i.e., caused by human activities).<br>Emissions of greenhouse gases this century will only cause warming this century.<br>Future heat waves are likely to be more frequent and more intense than those in the historic record.<br>Climate change will drive migration of animals, plants, bacteria, and viruses to new regions.<br>Climate change is likely to have negative effects on human health.<br>In Liarakou et al., 2010, 22 items, such as:<br>The greenhouse effect will not bring changes in the global food production.<br>Natural gas does not contribute to aggravating the greenhouse effect.<br>The ozone layer depletion exacerbated the greenhouse effect.	
**Alenda-Demoutiez, 2022**	People have different ideas about what causes climate change.<br>What about you, which of the following do you think is the main cause of climate change, or haven’t you heard enough to say?<br>Do you think climate change is making life in [country] better or worse, or haven’t you heard enough to say?	Human activity, like burning fuel and other activities that pollute the atmosphere, natural processes, both human activity and natural processes, non of these, don’t know/ haven’t heard enough;<br>Somewhat better, neither/no change/about the same, somewhat worse, much worse, don’t know/haven’t heard enough;<br>Yes, ordinary Ghanaians can do nothing at all, ordinary Ghanaians can do a little bit, ordinary Ghanaians can do a lot, don’t know

	Do you think that climate change needs to be stopped?<br>[If yes]<br>How much do you think that ordinary Ghanaians can do to stop<br>climate change?	
**Asgarizadeh et al., 2023: Das et al., 2022; Escoz Roldan et al., 2019, Fernandez et al., 2023, Flora et al., 2014; Higuchi et al., 2018; Rahman et al., 2020;**<br>**Stevenson et al., 2014, Stevenson et al., 2016**	11 correct-incorrect questions about physical knowledge, causes, consequences and action-related knowledge of climate change, for example “At the same quantity, CO_2 is more harmful to the climate than methane” and “climate change is mainly caused by human activities”, “nuclear power plants emit CO” during their operation”<br>In Das et al., 2022:<br>1 Is climate change both natural and man-made?<br>2 Do you think greenhouse gases cause climate change?<br>3 Can you name two major sources that release GHGs?<br>4 Is stubble burning by farmers is having negative impact on climate?<br>5 Is use of some electronic appliances (e.g., Fridge, Air conditioners) is contributing to climate change?<br>6 Does improper dumping and burning of household and farm waste cause climate change?<br>7 Are you aware that increased carbon dioxide will catalyse the process of climate change?<br>8 Does burning of fossil fuels cause climate change?<br>9 Do you think rise in sea level is because of climate change?<br>10 Is there any shift in seasonality of many crops due to climate change in past 10–15 years?<br>In another study (Escoz Roldan et al., 2019) 35 similar questions, such as:<br>The greenhouse effect is a natural phenomenon. (Correct)<br>The majority of the greenhouse gases present in the atmosphere come from natural sources. (Correct)<br>CO2 is the main gas responsible for climate change. (Correct)	Correct or incorrect

The increase in meat consumption contributes to climate change. (Correct)

Whenever using coal, oil, or gas, we contribute to climate change. (Correct)

Climate change is caused by human activity. (Correct)

Climate change is the result of natural climate variability. (Incorrect)

CO2 is a natural component of the atmosphere. (Correct)

There is scientific consensus when considering human activity as the main cause of climate change. (Correct)

The greenhouse effect is caused by human activity. (Incorrect);

In Fernandez et al., 2023 36 questions, for example:

Climate and climate change science

Climate and weather are the same.

The climate system consists of five major components: atmosphere, hydrosphere, lithosphere, cryosphere and biosphere. They act in a connected manner establishing a flow of matter and energy.

Since records have been kept, climate in Spain has always been the same.

Climate changes have contributed to the expansion and collapse of great civilizations.

Climate cannot be altered by historical factors.

In the last centuries, even in the last thousands of years, the area covered by ice in the northern hemisphere remained unchanged.

Current climate change is mostly caused by natural variations, such as changes in solar radiation or volcanic eruptions.

The last major climate change in Spain occurred in the 17th century.

Since the formation of our planet, climate remained practically unchanged until the last 10.000 years.

In Flora et al., 2014 authors showed only correct answers. The questions were:

The “greenhouse effect” refers to… (The way certain gases in the Earth’s atmosphere trap heat.)
Check ALL of the following that are greenhouse gasses. (Carbon dioxide, Methane)

By studying ice cores, scientists have shown that when carbon dioxide levels in the atmosphere are higher… (The Earth’s average temperature is higher.)

Check ALL of the following that are fossil fuels. (Coal, Oil, Natural gas)

What is the main gas produced by burning fossil fuels? (Carbon dioxide)

How does the US compare to the rest of the world in its use of natural resources? (More)

Check the ONE impact NOT from global warming. (More earthquakes)

Which of the following naturally takes carbon dioxide out of the atmosphere? (Trees)

Which of the following is a source of methane? (Cow burps)

The amount of carbon dioxide in the atmosphere TODAY is: (Higher)

Higuchi et al., 2018 had 15 statements, such as:
K1/Carbon—The Amazon forest uses carbon from greenhouse gases for its growth
K2/Fire—Burning the forests makes a small contribution to the climate change in Brazil
K3/Agriculture—Climate changes alter the world’s agricultural production
K4/Population—The population explosion contributes to climate changes
K5/Neutralization—The Amazon forest neutralizes the polluting gases causing climate change
K6/Floods and Droughts—The floods and droughts in the Amazon are caused by climate change
K7/Rain cycle—The Amazon forest controls the cycle of rain for the whole planet

	K8/Fertilizers—The use of chemical fertilizers prevents climate change<br>K9/Weather—The variations and inconsistency of the world's weather are the result of climate change<br>K10/Consumption—The current model of production and consumption is the principal reason for climate change K11/Reforestation—Reforestation of the Amazon has the same role as maintaining standing forest.<br>In Rahman et al., 2020 the questions were:<br>Climate change<br>Have heard about climate change<br>Believe that climate is changing<br>Have good understanding and awareness about climate change<br>Biggest local climate change problem in Bangladesh<br>Increase in extreme weather (more heat/very cold/heavy or torrential rainfall)<br>River bank erosion<br>Floods<br>Droughts<br>Believe that changes in climate can affect numbers of vectors and dengue transmission.<br>In Stevenson et al., 2016, 19 statements were included, such as:<br>Burning oil, among other things, produces carbon dioxide (CO2).<br>Carbon dioxide (CO2) is a greenhouse gas.<br>Greenhouse gasses partly keep the Earth's heat from escaping into space.<br>Carbon dioxide (CO2) is harmful to plants<br>The ozone hole is the main cause of the greenhouse effect.<br>At the same quantity, carbon dioxide (CO2) is more harmful to the climate than methane.	
**Asshoff et al., 2021, Bedford, 2016, Borhan & Ismail, 2011, Bozoglu et al., 2022, Bremer & Linnenluecke, 2017,**	23 items of multiple-choice instrument, for example:<br>When the concentration of CO_2 increases…<br>…the stomata increasingly close.<br>…the plant dies, because CO_2 is toxic.<br>…the stomata open for a short time.	F (false) or T (true), or Yes or No answers to True of False;<br>In Meira-Cartea et al., 2018 the true/false response options were in a form of a Likert scale from 1 (absolutely true) to 4 (absolutely false);<br>In Walker & McNeal, 2013 from 1 (definitely false) to 4 (definitely true)

**Connor et al., 2022, Dijsktra & Goedhart, 2012, Fischer et al., 2019, Fischer & Said, 2021; Gazzaz & Aldeseet, 2021; Hu et al., 2017; Hurst Loo & Walker, 2023; Meira-Cartea et al., 2018; Mumpower et al., 2016; Nussbaum et al., 2015; Pan et al., 2023; Thaller & Brudermann, 2020; Tremoliere & Djeriouat, 2021; Walker & McNeal, 2013; Wang et al., 2022**	…this has no effect on the opening and closing of the stomata.<br>In Bedford, 2016 statements such as:<br>Which of the following three statements do you think is more accurate? Scientists use the<br>term *greenhouse effect* to describe. . .<br>The heat-trapping properties of certain gases, such as carbon dioxide or CO2 (T)<br>A hole in the Earth's ozone layer, which allows more sunlight to get through<br>The warming effect of pavement and cities<br>Don't know;<br><br>Which of the following possible changes would, if it happened, do the most to raise sea<br>levels?<br>Melting of land ice in Greenland and the Antarctic (T)<br>Melting of glaciers in the Himalaya and Alaska<br>Melting of sea<br>Don't know;<br>In Borhan & Ismail, 2011:<br>52 items about causes for climate change in form of scientific and idiosyncratic statements, for example:<br>Increase CO2 volume in air composition<br>Gas from artificial fertilizers<br>Rainforest depletion<br>Eating meats<br>Too much CFC volume in air composition<br>Rotting waste<br>Use of heating & cooling systems in house<br>Too much ozone near the ground<br>Sunrays cannot escape from the earth<br>In Bozoglu et al., 2022 40 items about climate change, for example:<br>Cc will increase the dependency of farmers on agricultural support. (True)

Government has taken enough precautions against Cc in Turkey. (False)
It is necessary to provide assurance for reconstruction of agricultural land. (True)
It is necessary to provide assurance for reconstruction of agricultural land. (True)
It is necessary to provide assurance for reconstruction of agricultural land. (True)
It is necessary to provide assurance for reconstruction of agricultural land. (True)
It is necessary to provide assurance for reconstruction of agricultural land. (True)
Food standards need to be updated due to CC. (True)
It is not easy to struggle effectively with CC because people are attracted to different directions. (True)
The activities of one person do not make any difference in the struggle with CC. (False)
If every individual fulfills his or her responsibilities, CC problem can be ended. (True)
In Connor et al., 2022:
8 items:
Burning oil, amongst other things, produces carbon dioxide.
Carbon dioxide is harmful to plants.
At the same quantity, carbon dioxide is more harmful to the climate than methane.
For the next decades, the majority of climate scientists expect a warmer climate that increases the melting of polar ice, which will lead to an overall rise of the sea level.
For the next decades, the majority of climate scientists expect an increase in extreme events, such as droughts, floods and storms.
For the next decades, the majority of climate scientists expect a warmer climate to increase water evaporation, which will lead to an overall decrease of the sea level.
For the next decades, the majority of climate scientists expect the climate to change evenly all over the world.

∗Carbon dioxide, methane, and nitrous oxide are greenhouse gases that contribute to climate change?
In Dijsktra & Goedhart, 2012:
The most of the current climate change is due to greenhouse gases generated by human activity. If my city will have a heat wave this summer, it means climate is changing.
Climate change is only defined as the rising of temperature of the earth's surface.
Climate change is a result of the ozone layer becoming thinner. Climate change is partly caused by the increase in the emission of heavy metals.
Rise in sea level and drought are some of the consequences of climate change.
There is a direct link between climate change and skin cancer. The ocean can absorb CO2 emitted by humans.
Because of climate change, an oxygen deficiency can arise. Because of climate change, the water in seas and oceans will expand.
The acidification of the forest is a result of climate change. Because of climate change, certain plants and animals may become extinct.
In Fischer et al., 2019 8 items:
Science says that…
(1) The global average temperature in the air has increased approx. 3.1 °C in the past 100 years. (False)
(7) The 1990s was the warmest decade during the past 100 years. (False)
(2) The global change in temperature in the past 100 years is the largest during the past 1,000 years. (True)
(8) Climate change is mainly caused by a natural variation in sunbeam and volcanic eruption. (False)
(3) Carbon dioxide concentration in the atmosphere has increased more than 30% during the past 250 years. (True)
(4) The increase of greenhouse gases is mainly caused by human activities. (True)

(9) The blanket of snow in the Northern Hemisphere has decreased approximately 10% since the 1960s. (True)
(6) An increasing amount of greenhouse gases increases the risk of more UV-radiation and therefore a larger risk of skin cancer. (False)
In Fischer and Said, 2021, are also 8 items:
Science says that…
(1) The global average temperature in the air has increased approx. 3.1 °C in the past 100 years. (False)
(7) The 1990s was the warmest decade during the past 100 years. (False)
(2) The global change in temperature in the past 100 years is the largest during the past 1,000 years. (True)
(8) Climate change is mainly caused by a natural variation in sunbeam and volcanic eruption. (False)
(3) Carbon dioxide concentration in the atmosphere has increased more than 30% during the past 250 years. (True)
(4) The increase of greenhouse gases is mainly caused by human activities. (True)
(9) The blanket of snow in the Northern Hemisphere has decreased approximately 10% since the 1960s. (True)
(6) An increasing amount of greenhouse gases increases the risk of more UV-radiation and therefore a larger risk of skin cancer. (False)
In Gazzaz & Aldeset, 2021 there were 30 questions, such as:
Climate change is real, that is, it is taking place (True)
Manifestations of climate change differ from one climatic region to another (True)
Climate change is inevitable because of the nature and style of the modern life (True)
Climate change is nothing else than natural fluctuation in temperatures of the Earth (False)
The human community can control climate change (True)
Overall, climate change is bad; it is more harmful than beneficial (True)
Climate change is the tangible, long-term change in weather variables that is associated with the increases

in the concentrations of the green house gases in the atmosphere (True)
Scientific evidence on climate change is non-reliable (False)
The average minimum and maximum temperatures never increased anywhere on the Earth since 1900 (False)
It is too late for the human community to do anything to stop climate change or reduce it (False)
Hu et al., 2017 included 8 true/false statements:
Burning oil produces CO_2.
CO_2 is harmful to plants.
Nuclear power plants emit CO_2 during operation.
At the same quantity, CO_2 is more harmful to the climate than methane.
The global CO_2 concentration in the atmosphere has increased during the past 250 years.
Climate change is mainly caused by human activities.
The last century's global increase in temperature was the largest during the past 1000 years.
Today’s global CO_2 concentration in the atmosphere has already occurred in the past 650,000 years.
Hurst Loo & Walker, 2023 included 12 statements, for example:
Climate change causes knowledge
The global CO_2 concentration in the atmosphere has increased during the past 250 years.
Today’s global CO_2 concentration in the atmosphere has already occurred in the past 650,000 years.
Climate change is mainly caused by human activities.
The last century’s global increase in temperature was the largest during the past 1000 years.
Climate change consequences knowledge
For the next decades, the majority of climate scientists expect ...
a warmer climate to increase the melting of polar ice, which will lead to an overall rise of the sea level.
an increase in extreme events, such as droughts, floods, and storms

a warmer climate to increase water evaporation, which will lead to an overall decrease of the sea level.

In Meira-Cartea et al., 2018 there were 32 items, such as:

1. The greenhouse effect is a natural phenomenon.
2. The majority of greenhouse gases present in the atmosphere are from natural sources.
3. CO2 is the main gas responsible for CC.
4. Increased meat consumption contributes to CC.
5. Every time coal, petrol or gas is used we contribute to CC.
6. CC is caused by human activity.
7. CC is the result of natural climate variability.
8. CO2 is a natural component of the atmosphere.
9. There is scientific consensus that considers human activity to be the main cause of CC.
10. The greenhouse effect is caused by human activity.

In Mumpower et al., 2016, there were 10 true/false items, for example:

(a) the major cause of increased atmospheric concentration of greenhouse gases is human burning of fossil fuels (T);

(b) nitrous oxide is a greenhouse gas (T);

(c) aerosols are airborne particles that are known to contribute to the formation of clouds and precipitation (T);

(d) the greenhouse effect refers to gases in the atmosphere that trap heat (T);

(e) climate often changes from year to year (F);

(f) ocean currents carry heat from the equator to the north and south poles (T);

(g) the US emits the largest total amount of carbon dioxide (F);

(h) the energy in fossil fuels originally came from the fossilized remains of plants and animals (T);

(i) the average yearly temperature of the Earth's surface is currently above 65°F (F);

(j) the Earth’s climate is warmer now than it has ever been before (F).

Nussbaum et al., 2016 had 7 true/false questions:

1. The greenhouse effect and the ozone layer are basically the same. T or *F
2. Climate change and changes in weather are the same. T or *F
3. Over the past 100 years global temperatures have increased. *T or F
4. Humans cannot survive on Earth without the greenhouse effect. *T or F
5. Human activities produce greenhouse gases. *T or F
6. Requiring everyone in the community to cover their swimming pool would save very little water. T or *F
7. Resorts and casinos waste a large amount of water. T or *F

Pan et al., 2023 included 30 statements, for example:

1. The last century's global increase in temperature was the largest during the past 1,000 years. T
2. The Sun is the primary source of energy for Earth ’s climate system. T
3. In addition to CO2, water vapor, methane are also greenhouse gases. T
4. Greenhouse gases cannot be removed naturally. F
5. The presence of small amounts of greenhouse gases helps to sustains life in the Earth. T
6. Climate change will have consequences for the nature and human lives. T
7. The input of energy from the sun is a major cause of global warming. F
8. Climate varies over space and time through man-made instead of natural processes. F
9. Climate change is mainly caused by human activity. T
10. Life (microbes, plants, and animals and humans) is a major driver of the global carbon cycle. T

	In Thaller & Brudermann, 2020, 10 items were included:<br>Water vapor is a greenhouse gas.<br>A diesel vehicle generates more CO2 emissions per person and kilometer than a comparable petrol vehicle.<br>The ozone hole is the main cause of the greenhouse effect.<br>Without humans there would be no greenhouse effect.<br>The 1990s were the warmest decade of the 20th century.<br>CO2 is more harmful to the climate than the same amount of methane.<br>In the last century, warming in Austria was significantly lower than the global average.<br>The global rise in temperature in the last century was the biggest within the last 1.000 years.<br>The production of 1 kg of pork produces more greenhouse gas emissions than the same amount of wheat.<br>If the current greenhouse gas content in the atmosphere was stabilized, the climate would nevertheless continue to warm up for at least 100 years.<br>In Tremoliere & Djeriouat, 2021, 19 statements were included, such as:<br>The global CO2 concentration in the atmosphere has increased during the past 250 years.<br>The increase of greenhouse gases is mainly caused by human activities.<br>With a high probability, the increase of CO2 is the main cause of climate change.<br>Climate change is mainly caused by natural variations (such as changes in solar radiation intensity and volcanic eruptions).<br>The last century’s global increase in temperature was the largest during the past 1,000 years.	

	The ‘90s were globally the warmest decade during the past century.<br>Today’s global CO2 concentration in the atmosphere already occurred in the past 650,000 years.<br>In Walker & McNeal, 2013 27 statements were included, for example:<br>A warming of the Earth can cause...<br>1. Disruptions in agriculture<br>2. Changes in animal migration patterns<br>3. Changes in regional environments<br>4. More UV radiation<br>5. An increase in the size of the ozone hole<br>6. Sea level rise<br>7. Glaciers to melt<br>8. Arctic ice to melt<br>9. Coral reef die off<br>10. Flooding of New York City<br>11. Increased homeland security threats	
**Banwel et al., 2020**	2. Do you think that climate change is affecting your locality?<br>3. How important is climate change on a scale of 1-5 (5 being very important)<br>4. Do you believe that climate change is caused by human activity?<br>5. What causes climate change?<br>6. Has your work been affected by extreme weather phenomena/ processes?<br>7. If yes, on a scale of 1-5, how much has your work been affected?<br>8. Do you usually receive information about the weather forecast?	Answers for 2: a. Yes, b. No<br>for 3.: scale from 1 do 5 (very important)<br>for 4.: a. Yes, b. No<br>for 5.: a. industrial activity, b. deforestation, c. burning oil, d. increase in livestock activity, e. other_____<br>for 6.: a. Yes, b. No<br>for 7.: scale from 1 do 5<br>for 8.: a. Yes, b. No
**Boon, 2016**	7 multiple-choice questions, such as:<br>1. Climate change is caused by…<br>2. The ozone layer has been mainly depleted by…<br>3. The biggest environmental threat to Australian farmland as a result of climate change is..	Please circle the answer you think is most appropriate in each question below.<br>1. :<br>a) a hole in the earth’s atmosphere<br>b) natural climate fluctuations<br>c) increased cloud cover<br>d) increased carbon emissions

	4. Some water, a small amount of soil, a few green aquatic plants and a fish were placed in a large bottle. The bottle was sealed to prevent the exchange of gases and other materials between its contents and the outside. The bottle was placed in a window to receive light during the daytime. Is carbon dioxide produced by the plants?<br>5. Greenhouse gases in the lower atmosphere (troposphere) absorb:<br>6. The major source of anthropogenic carbon emissions comes from:<br>7. The major human impact on the water cycle is:	e) solar activity<br>2.:<br>a) burning of fossil fuels<br>b) pollution from garbage tips<br>c) the release of CFC's into the atmosphere<br>d) the increasing temperature of the sun's rays<br>3.:<br>a) soil salinity<br>b) land clearing<br>c) drought<br>d) pesticides<br>4.:<br>a) Yes, but it is produced only at night when the plants can no longer carry on photosynthesis.<br>b) Yes, it is produced all the time as a result of cellular respiration.<br>c) No, it is a waste product of animals only.<br>d) No, plants take in only the waste products exhaled by animals.<br>e) No, plants only produce oxygen<br>5. :<br>a) incoming ultraviolet radiation<br>b) infrared radiation emitted by the earth's land surfaces and oceans<br>c) incoming solar radiation reflected by clouds<br>d) incoming solar radiation across the entire electromagnetic spectrum<br>6. :<br>a) using coal to generate electricity<br>b) burning fossil fuels, industrial processes<br>c) increased run-off of nutrients from farmland<br>d) increased populations of animals and humans breathing out carbon dioxide and producing methane gas<br>7. :<br>a) increasing population breathing out more carbon dioxide and water vapour<br>b) acidifying rain water by the burning of fossil fuels<br>c) run off from industry, agriculture and sewage which dissolve in water and pollutes it<br>d) humans do not have an impact on the water cycle; it is governed by the sun's energy, circulating it through its phases<br>e) over use causing water to run out
**Chuvieco et al., 2021**	2 items:<br>1. Causes of climate change from five choices<br>2. Rank the importance of natural factors to CC	Five choices for 1. question:<br>1) deterioration of the ozone layer,<br>2) variations of solar radiation,<br>3) aerosols,<br>4) greenhouse gases (GHG) and<br>5) "Don't know".<br>In the second, the importance was measured between 1 (very low) and 5 (very high).

**Di Gusto et al., 2018**	15 questions, for example:<br>1. Climate refers to the average weather conditions in a place over a period of years.<br>2. Climate change includes humidity and wind, but weather does not.<br>3. From 1880 to the present, which of the following years was the hottest?<br>4. Which of the below is not a predicted impact from global warming?<br>5. What factors cause the 'greenhouse effect'?<br>6. Which radiation below could be absorbed by the greenhouse gases in the atmosphere<br>7. Which of the below is the most potent greenhouse gas?<br>8. Which of these activities releases the most carbon dioxide into the atmosphere?<br>9. Which of the gases below associated with climate change has the fastest growth rate?<br>10. Which of the following methods of transportation consumes more energy per person per kilometer?	Answers for 2.:<br>a. 1<br>b. 2<br>c. 3<br>d. 1 and 2<br>for 3.:<br>a. 1970<br>b. 1934<br>c. 1998<br>d. 2015<br>for 4.:<br>a. higher temperatures globally<br>b. more extreme weather<br>c. extinction of some species<br>d. rise in mean sea level<br>e. increased acid rain<br>for 5.:<br>a. The chemical reactions of greenhouse gases produce extra heat<br>b. There are too many greenhouses spread across the surface of the earth<br>c. Carbon dioxide and other gases retain heat in the atmosphere<br>d. Sulfides are continuously emitted by chimneys and volcanos<br>e. Gases produced by human activities cause heat to leave the Earth's atmosphere<br>for 6.:<br>a. visible light<br>b. ultraviolet<br>c. short wave<br>d. infrared<br>for 7.:<br>a. methane<br>b. carbon dioxide<br>c. ozone<br>d. argon<br>for 8.:<br>a. fossil fuel combustion<br>b deforestation<br>c ozone loss<br>d acid rain<br>e agriculture<br>for 9.:<br>a water vapor<br>b methane

		c chlorofluorocarbons<br>d carbon dioxide<br>e oxygen<br>for 10.:<br>a rail transportation<br>b personal car (single traveler)<br>c bus<br>d car sharing (driver with other passengers)
**Ebuehi & Olusanya, 2013, Helbling et al., 2021**	Items such as:<br>Awareness on climate change<br>Source of information<br>Definition of climate change<br>Climate change is a global problem<br>Cause of climate change<br>Number of greenhouse gases etc.<br>Like the study before, Helbling et al., 2021 had 3 questions, first of which was in a form of Yes/No, with a follow up questions:<br>Have you heard about climate change or haven't you had the chance to hear about this yet?<br>(If Yes) What does the phrase "climate change" mean to you?<br>Negative changes in the weather like more droughts, floods or extreme heat,<br>Positive changes in the weather like better rainfall patterns or longer growing seasons, Other changes in weather patterns<br>(If Yes) Do you think climate change is making life in [in your country] better or worse, or haven't you heard enough to say?	Yes or No;<br>Media, People, School, Others;<br>Change in the properties of climate, change in volume of some gases, change in the pattern of rainfall, change in the temperature of the earth, all of the above, do not know;<br>Yes, no;<br>Human, natural, both;<br>None, one, two, three, four, five;<br>In Helbling et al., 2021:<br>Yes or No with a follow up of 5-point scale from 1 (much better) to 5 (much worse),
**Geiger et al., 2014**	36 items who measured environmental knowledge, for example:<br>Which of the following phenomena is the principal cause for rising temperature of the planet in the last 20 years?<br>2. Which if these energy forms is not	Multiple-choice

	renewable?<br>3. What is the problem with CO_2?<br>4. What is the carbon footprint of a product?<br>5. How many meters would the sea level rise if all the polar ice caps were to melt completely?<br>6. Doubling the CO_2 concentration in the atmosphere. the mean global temperature would rise in long term…<br>7. Which of the following practices is accepted in ecological agriculture?<br>8. Which of the following health problems cannot stem from heavy metals?<br>9. What is the most prominent cause of the growing pollution of ground water with Nitrate?<br>10. Which of the following statements (concerning ground water) is correct?	
**Gutierrez et al., 2022**	Objective knowledge was measured via 76 items, grouped in seven Essential Principles of Climate Science:<br>1. the sun is the primary source of energy for Earth's climate system. (2 items)<br>2. climate is regulated by complex interactions among components of the Earth system. (6 items)<br>3. life on Earth depends on, is shaped by, and affects climate. (1 items)<br>4. climate varies over space and time through both natural and man-made processes. (16 items)<br>5. our understanding of the climate system is improved through observations, theoretical studies, and modeling. (2 items)<br>6. human activities are impacting the climate system. (43 items)<br>7. climate change will have consequences for the Earth system and human lives. (6 items)	Correct/Incorrect
**Hallar et al., 2011**	Quiz with 6 items:<br>1. Which of these is an example of climate?	Answers for 1.:<br>Windy day<br>Rainy day

	2. Where do greenhouse gases trap energy?<br>3. Which one of these is a greenhouse gas?<br>4. Which of these is a way to measure the weather?<br>5. What gas makes up most of the atmosphere?<br>6. Why did the pressure go down when we were at the top of the gondola?	Hot summer<br>Sunny day<br>for 2.:<br>In the atmosphere<br>In the mountain<br>In outer space<br>In the soil<br>for 3.:<br>Oxygen<br>Carbon dioxide<br>Wind<br>Sulfur dioxide<br>for 4.:<br>Temperature<br>Pressure<br>Relative humidity<br>All of the above<br>What gas makes up most of for 5.:<br>Nitrogen<br>Ozone<br>Carbon dioxide<br>Hydrogen<br>for 6.:<br>The temperature went down<br>The wind speed went up<br>The snow depth went up<br>We went up in altitude
**Javeline et al., 2019**	3 questions about climate change causes:<br>1. To the best of your knowledge, which, if any, of the following are major contributors to climate change?<br>2. If nothing is done to prevent it, how likely do you think it is that global warming will cause any of the following during the next 100 years?<br>3. When oceans water gets warmer, does its volume expand, contract, or stay the same?	Answers option for 1.:<br>a. Destruction of forests<br>b. Carbon dioxide emissions<br>c. The hole in the ozone layer<br>d. Emissions from power plants<br>e. Emissions from transportation<br>f. Use of gas, electricity by industry<br>g. Use of gas, electricity in homes<br>h. Use of mobile phones<br>response options: no/yes<br>for 2.:<br>a. Rise in the world’s sea level<br>b. More damaging storms

		c. More frequent flooding<br>response options: not at all likely/not very likely/somewhat likely/very likely<br>and for 3. response options were contract/stay the same/ expand
**Jurek et al., 2022; Karpudewan & Mohd Ali Khan, 2017; McNeill & Vughn, 2012; Schollaert Uz et al., 2014; Bodzin et al., 2014; Kolenaty et al., 2022**	6 questions about causes and consequences of climate change In Jurek et al., 2022:<br>State the factors which cause climate change.<br>State the consequences of climate change.<br>Factual knowledge of the greenhouse gases:<br>State the gases that are involved in the greenhouse effect.<br>Try to order the above gases from the one that plays the greatest part in the creation of the greenhouse effect to the one that plays the least.<br>Principle and underlying logic of greenhouse effect:<br>On what principle does the greenhouse effect work?<br>Take time to consider and imagine what the Earth would be like if there were no greenhouse gases in the atmosphere: describe it briefly.<br>Karpudewan and Mohd Ali Khan (2017) included 5 open-ended items, for example:<br>What are the main causes for climate change?<br>How can we reduce the rate of climate change?<br>McNeill & Vughn, 2012 had 1 open-ended question:<br>What are three human behaviors that impact climate change? Why?<br>In Schollaert Uz et al., 2014, 1 open-ended question were included:<br>What is the name of the microscopic plant in the ocean that forms the base of the food web and creates half of all the oxygen we breathe?<br>Bodzin et al., 2014 had 3 questions:<br>Describe at least four different types of human activities that are causing the long-term increase of carbon dioxide levels over the last 100 years?<br>Describe at least four different things that you can do to reduce or prevent further emissions of carbon dioxide into the atmosphere?	Open-ended

	What would it actually take for all the people on our planet to lower the levels of carbon dioxide in the atmosphere?	
**Kumar et al., 2023**	3 questions divided into 3 groups:<br>Knowledge about the cause of global warming:<br>Which of the following factors is currently the biggest contributor to global warming?<br>Knowledge about the mechanism of global warming:<br>Which of the following gases in the atmosphere is considered a greenhouse gas?<br>Knowledge about the effects of global warming:<br>By how much could sea levels rise in the next 30 years as a result of global warming?	Responses for 1.: a) Human activities b) Natural changes in the environment c) None of the above because global warming isn't happening d) Other e) Don 't know<br>For 2.: a) Oxygen b) Hydrogen c) Carbon dioxide d) Don 't know<br>For 3.: a) Sea levels will not rise b) From 20 cm to 1 m c) From 4 m to 10 m d) More than 10 m e) Don 't know
**Adu-Boateng et al., 2023; Liu et al., 2022; Klapp & Bouvier-Brown, 2021; McNeill & Vughn, 2012; Ngo et al., 2020; Nussbaum et al., 2016;<br>Schollaert Uz et al., 2014; Seebauer, 2014; Siegner & Stapert, 2020; Tolppanen et al., 2023; Tranter, 2020; Tranter, 2021; Tranter et al., 2020; Wang et al., 2020; Yeh et al., 2024; Bodzin et al., 2014; Player et al., 2023; Harker-Schuch, 2020; DeCamp, 2024; Carrol Steward et al., 2023; Anyanwu & Le Grange, 2017; Karpudewan et al., 2014**	In Adu-Boateng et al., 2023, questions such as:<br>Have you heard about climate change? If yes, hoy do you understand climate change?<br>11 multiple choice questions (Liu et al., 2022):<br>1. The greenhouse effect can best be described as...<br>2. Which of the following is NOT a greenhouse gas?<br>3. Which is the most abundant greenhouse gas?<br>4. Average global temperatures from thousands of years ago can be determined by measuring which of the following from a glacial ice core?<br>5. Complete the following statement by choosing one response: The rate of climate change can best be slowed by ________.<br>In Klapp & Bouvier-Brown, 2021there were 31 questions, such as:<br>1. The dominant greenhouse gases..<br>2. The greenshouse effect is made worse<br>...by too much ozone near tthe grount<br>....besause the Sun's rays cannor escabe the Earth etc.<br>McNeill & Vughn, 2012 had 6 multiple-choice questions, such as:<br>1. Climate change…	In Adu-Boateng et al., 2023, answers for 1.:<br>a. changes in rainfall patterns<br>b. changes in temperature patterns<br>c. changes in rainfall and temperature patterns<br>d. act of God<br>e. I don't know what it means<br>In Liu et al., 2022, for 1.:<br>(a) The same thing as global warming<br>(b) Pollution related to acid rain<br>(c) An increasing of the temperature of the planet<br>(d) Damage to the ozone layer<br>2. :<br>(a) Carbon dioxide (CO_2)<br>(b) Chlorofluorocarbons (CFCs)<br>(c) Water vapor (H_2O)<br>(d) Oxygen (O_2)<br>(e) Methane (CH_4)<br>3.:<br>(a) Carbon dioxide (CO_2)<br>(b) Hydrogen (H_2)<br>(c) Methane (CH_4)<br>(d) Nitrogen (N_2)<br>(e) Water vapor<br>4:<br>(a) The half-life of radioactive water.<br>(b) The size of ice crystals.

	2. Greenhouse gases in the Earth's atmosphere trap:<br>3. Carbon sequestration is… etc.<br>In Ngo et al., 2020, there were 2 multiple-choice questions:<br>What do you know about the causes and impacts of floods?<br>What do you know about the impacts of climate change?<br>Nussbaum et al., 2016 had 13 multiple-choice questions, for example:<br>1. There are ways to conserve water at home. Some ways save more water than<br>others. Which of the following is the best way to conserve water at home?<br>2. There are ways to conserve water within your community. Some ways save more water than others. Which of the following is the best way to conserve water within your community?<br>3. Which of the following options would save the most water? etc.<br>In Schollaert Uz et al., 2014, 8 multiple-choice questions were included, for example:<br>What do satellites measure that is used to determine how much plant life is in the ocean's surface?<br>What influences how much phytoplankton grows?<br>What gives plants their color and can be used to estimate phytoplankton abundance?<br>In Seebauer, 2014 22 quiz questions were included, such as:<br>What effect does the greenhouse effect describe?<br>Carbon dioxide is responsible for approximately:<br>Which of the following gases has the strongest influence on the greenhouse effect per ton?<br>In Siegner & Stapert, 2020, 16 questions, for example:<br>What is causing the global warming?<br>What is the current CO_2 concentration in the atmosphere, in parts per million (ppm)? etc.	(c) The percentage of atmospheric nitrogen.<br>(d) The ratio of deuterium to hydrogen<br>5. :<br>(a) "going green" and recycling more<br>(b) switching from fossil fuels to renewable energy sources<br>(c) driving less or driving smaller vehicles<br>(d) reducing pollution<br>In Klapp & Bouvier-Brown, 2021, options for 1.:<br>a) do not occur naturally in the atmosphere.<br>b) are necessary for life as we know it on Earth.<br>c) did not exist in pre-industrial times, for 2 and 3.:<br>b) I think this is right<br>c) I don't know<br>d) I think is this wrong<br>e) I am sure this is wrong<br>In McNeill & Vughn, 2012, response options for 1. were:<br>a. is only caused by human activities<br>b. explains a previous summer's heat wave<br>c. is a change in long-term weather patterns<br>d. explains the decrease in available fossil fuels,<br>for 2:<br>a. heat, but not visible and ultraviolet light<br>b. visible light, but not heat and ultraviolet light<br>c. ultraviolet light, but not heat and visible light<br>d. heat, visible and ultraviolet light<br>for 3.:<br>a. The production of carbon dioxide by cars<br>b. The amount of carbon that is produced by burning one tree<br>c. The amount of carbon dioxide that is in the Earth's atmosphere<br>d. The removal and storage of carbon from the Earth's atmosphere<br>Response options in Nussbaum et al., 2016 for 1.:<br>a) Stop washing the family car at home.<br>b) Replace your old washing machine with an energy and water-efficient one.<br>c) Install low-flow faucets in your home.<br>d) *Replace lawns with desert landscape.<br>For 2.:<br>a) Reduce watering golf courses in the area.<br>b) Shut down any outside casino fountains.<br>c) *Raise water prices.<br>d) Require everyone in the community to cover their swimming pools.<br>For 3.:<br>a) Planting trees.<br>b) Not washing cars at home.

In Tolppanen et al., 2023 the questions and response options were similar:<br>According to climate scientists, how has the amount of carbon dioxide in the atmosphere changed since the start of the Industrial Revolution 150 years ago?<br>According to climate scientists, which of the following statements about global warming over the past 50 years is most accurate?<br>Which is the best description of the differences between climate and weather?<br>Which of the following contributes to the transfer of thermal energy from place to place around the Earth?<br>How does sunlight affect temperature on Earth?<br>Which of the following will occur if the amount of ice floating in the ocean decreases?<br>Which of the following would most likely occur if the oceans stopped absorbing carbon dioxide?<br>Which is the best definition of a positive feedback loop in the climate system?<br>Which of the following is the best definition of a greenhouse gas?<br>How much incoming sunlight do greenhouse gases absorb?<br>Questions in Tranter, 2020, Tranter, 2021 and Tranter et al., 2020:<br>Which of the following best describes the North Pole?<br>Which of the following best describes the South Pole?<br>Which of the following possible changes would, if it happened, do the most to raise sea levels?<br>In Wang et al., 2020, there were 6 questions, such as:<br>Which of the following is the best definition of a greenhouse gas?<br>How has the amount of carbon dioxide in the atmosphere changed since the start of the Industrial Revolution 150 years ago?<br>Which is the best description of the differences between climate and weather?	c) *Using grey water systems.<br>d) Taking shorter showers.<br>In Schollaert Uz et al., 2014, answers for 1.:<br>a) the color of the ocean<br>b) the temperature of the ocean<br>c) how rough it looks<br>d) all of the above<br>for 2.:<br>a) sunlight and nutrients<br>b) seasons<br>c) El Nin˜o and La Nin˜a<br>d) all of the above<br>for 3.:<br>a) chromatin<br>b) chlorophyll<br>c) microfilm<br>d) all of the above<br>In Seebauser, 2014, response options for 1.:<br>The protection from solar radiation by the Earth's ozone layer<br>Gases in the atmosphere that trap heat<br>The reflection of sunlight by clouds<br>How plants grow<br>For 2.:<br>60% of the overall emissions of greenhouse gases<br>70% of the overall emissions of greenhouse gases<br>80% of the overall emissions of greenhouse gases<br>90% of the overall emissions of greenhouse gases<br>For 3.:<br>Nitrous oxide/laughing gas<br>Methane<br>Carbon dioxide<br>Chlorofluorocarbon/CFC<br>In Siegner & Stapert, 2020, response options for 1.:<br>a. The sun moving closer to the Earth.<br>b. Melting glaciers<br>c. The added or extra greenhouse effect due to increased concentration of heat-trapping gases from<br>human activities that prevent solar radiation from leaving Earth's atmosphere.<br>d. The increase in Nitrogen and Oxygen as a percentage of the total gases in Earth's atmosphere.<br>For 2.:<br>a. 280 ppm<br>b. 400 ppm

Examples of questions in Yeh et al., 2024:<br>Which of the following is a phenomenon of climate change?<br>Which of the following gases does not contribute to the greenhouse effect?<br>Compared to pre-industrial levels, the current global warming is approximately how many degrees Celsius?<br>In Player et al., 2023 30 questions were included, for example:<br>Which statement is correct?<br>What causes wind?<br>On average, how long does it take to form 10cm of fertile soil?<br>What is an ecological niche?<br>In Harker-Schuch, 2020, there were 27 questions about climate changes similar to previous studies, such as:<br>Which of the following would most likely occur if the oceans stopped absorbing carbon dioxide?<br>Which is the best description of the differences between climate and weather?<br>Which of the following statements about global warming over the past 50 years is most accurate?<br>In Decamp, 2024 some questions were:<br>What is the greenhouse effect?<br>The difference between weather and climate is<br>Which of these changes can make Earth's temperature increase, generally speaking?<br>In Carrol Steward et al., 2023, example of questions are:<br>Which of the following is the best definition of a greenhouse gas?<br>How has the amount of carbon dioxide in the atmosphere changed since the start of the Industrial Revolution 150 years ago?<br>Which of the following contributes to the transfer of thermal energy from place to place around the Earth?<br>In Anyanwu & Le Grange, 2017, 15 questions such as:	c. 350 ppm<br>d. 500 ppm<br>Answers in Tranter, 2020, Tranter, 2021 and Tranter et al., 2020 for 1.:<br>Ice a few meters thick floating over a deep ocean<br>Ice more than a kilometer thick over land<br>A mainly rocky, mountainous landscape<br>Don't know<br>For 2.:<br>Ice a few meters thick floating over a deep ocean<br>Ice more than a kilometer thick over land<br>A mainly rocky, mountainous landscape<br>Don't know<br>For 3.:<br>Melting of Glaciers in the Himalayas and Alaska<br>Melting of Sea ice on the Arctic Ocean<br>Don't know<br>Answers in Wang et al., 2020 for 1.:<br>a. An atmospheric gas that is produced as plants grow.<br>b. An atmospheric gas that absorbs infrared radiation.<br>c. An atmospheric gas that produces acid rain.<br>d. An atmospheric gas that absorbs ultraviolet radiation.<br>e. I do not know.<br>For 2.:<br>a. The amount of carbon dioxide has remained the same.<br>b. The amount of carbon dioxide has decreased.<br>c. The amount of carbon dioxide has increased.<br>d. I do not know.<br>For 3.:<br>a. Climate does not change over time, and weather does change over time.<br>b. Climate changes over time, and weather does not change over time.<br>c. Climate changes over long periods of time, and weather changes over short periods of time.<br>d. Climate changes over short periods of time, and weather changes over long periods of time.<br>e. I do not know.<br>Response option in Yeh et al., 2024 for 1.:<br>(1) Yesterday's rainfall in Wenshan District of Taipei City broke the record.<br>(2) The average temperature in January in Puli has risen by 1.5 degrees Celsius over the past 50 years.<br>(3) Soil erosion in the Tseng Wen Reservoir watershed is getting more severe.<br>(4) The apple yield in Lishan has been decreasing in recent years.<br>For 2.:<br>(1) 0.5 degrees (2) 1 degree (3) 2 degrees (4) 3 degrees

	15 multiple choice questions such as:<br>1. One of these options is the most abundant greenhouse gas and also the most important contributor to natural greenhouse effect.<br>Or<br>2. The term global warming refers to<br>In Karpudewan et al., 2014, questions such as:<br>The ozone layer…<br>Which one of the following is the reason for your answer to the previous question?	For 3.:<br>(1) Organizing a Mid-Autumn Festival barbecue<br>(2) Using plastic bags while shopping<br>(3) Discharging wastewater<br>(4) Burning natural gas<br>Response options in Player et al., 2023 for 1.:<br>a) Groundwater is composed of surface water that seeps into the ground (rain, rivers, lakes)<br>b) Groundwater comes from deep geological layers<br>c) Pollutants from river and rainwater do not get to the groundwater<br>d) Groundwater is old and is no longer being formed<br>for 2.:<br>a) The movement of the clouds<br>b) Differences in temperature and air pressure in the atmosphere<br>c) The moon's gravity<br>d) Ocean currents<br>For 3.:<br>a) 10 years<br>b) 80 years<br>c) 500 years<br>d) 2000 years<br>for 4.:<br>a) The place where an animal species lives the longest throughout the year<br>b) The interaction of biotic and abiotic nature in a specific place<br>c) The place where animals return to have their offspring<br>d) The place where prey species typically hide<br>Responses in Harker-Schuch, 2020 for 1.:<br>Carbon dioxide in the atmosphere would remain the same.<br>b. Carbon dioxide in the atmosphere would increase.<br>c. Carbon dioxide in the atmosphere would decrease.<br>d. I do not know.<br>For 2.:<br>climate does not change over time, and weather does change over time.<br>b. climate changes over time, and weather does not change over time.<br>c. climate changes over long periods of time, and weather changes over short periods of time.<br>d. climate changes over short periods of time, and weather changes over long periods of time<br>for 3.:<br>Global warming over the past 50 years is slightly due to natural processes and mostly due to human activities.<br>b. Global warming over the past 50 years is mostly due to natural processes and slightly due to human activities.

c. Global warming over the past 50 years is about equally due to natural processes and human activities.
d. Global warming over the past 50 years has not occurred whether due to natural processes or human activities.
Answers in DeCamp, 2024 for 1.:
Certain gases in the atmosphere trap heat and warm the Earth *
b. Life on Earth 'exhales' gas that warms up the atmosphere
c. The tilt of the Earth changes the amount of solar energy the Earth receives
d. The Sun is putting out more radiant energy over time
for 2.:
Weather is what we expect based on years of data while climate is what is happening now
b. Weather is predictable but climate is not
c. Weather is a day-to-day event while climate is a consistent pattern over many years
for 3.:
Increased emission of radiation from the Sun.*
b. Increased ice in polar regions.
c. Increased greenhouse gases in the atmosphere.*
d. Increased reflectivity (albedo) of Earth's surface.
Responses options for Carrol Steward et al., 2023 for 1.:
An atmospheric gas that is produced as plants grow.
b. An atmospheric gas that absorbs radiation.
c. An atmospheric gas that produces acid rain.
d. An atmospheric gas that reduces ozone in Earth’s atmosphere.
For 2.:
The amount of carbon dioxide has remained the same.
b. The amount of carbon dioxide has decreased.
c. The amount of carbon dioxide has increased.
d. I do not know
for 3.:
The movement of ocean water but not the movement of air.
b. The movement of air but not the movement of ocean water.
c. Both the movement of ocean water and the movement of air.
d. Neither the movement of ocean water nor the movement of air.
Answers in Anyanwu & Le Grange, 2017:
Answers to 1.:
A. Methane
B. Carbon dioxide
C. Water vapour

		D. Nitrous oxide<br>Answers to 2.:<br>A. A high concentration of carbon dioxide in the lower atmosphere<br>B. Rapid changes in dynamics and processes of the climate system<br>C. Changes in global climate and local weather patterns<br>D. Increase in the average temperature of the Earth's atmosphere<br>Answers in Karpudewan et al., 2014 for 1:<br>a. protects the earth from acid rain<br>b. filters the ultraviolet (UV) rays of the sun<br>c. helps to keep the earth'2 temperature stable to make it livable.<br>For 2.:<br>a. The ozone layer absorbs the sun's ultraviolet (UV) rays, which is potentially damaging to life on the earth.<br>b. The ozone layer prevents sunrays to exit from the atmosphere, consequently keeps it warm enough to live.<br>c. The ozone layer works as a kind of shield, so it does not let acid rain to reach the earth's surface.
**McCright, 2010**	3 questions:<br>Timing of global warming.<br>Primary cause of global warming.<br>Scientific consensus on global warming.	Responses for 1.:<br>(0 = not yet begun to happen; 1 = already begun to happen),<br>For 2.: (0 = natural changes in the environment; 1 = effects of pollution from human activities)<br>And for 3.: (0 = most scientists believe global warming is not occuring or most scientists are unsure; 1 = most scientists believe global warming is occurring)
**Peterson & Kozlowski, 2024**	1. global Warming has exacerbated the frequency of extreme weather events such as droughts, floods, and storms.<br>2. Climate Change has led to increases in global temperature.<br>3. Climate Change has led to temperatures that melt polar ice caps.<br>4. Climate Change has been and continues to lead to global sea-level rise.<br>5. Human activities are directly responsible for increases in atmospheric greenhouse gases.<br>6. Climate Change is a global crisis.<br>7. Climate Change is causing loss of land and livelihood for people around the globe.	1-does not fit, 2-fits construct, 3- strong fit to construct

**Powers et al., 2021; DeWaters et al., 2014**	42 items about the cognitive scale with different types of questions. The objective knowledge was measured with multiple-choice questions, for example:<br>The difference between weather and climate is…<br>Which of the following is the most abundant greenhouse gas?<br>Which of the following is the greenhouse gas of most concern from a global warming point of view?<br>Earth's atmosphere is warmer than it would be without a greenhouse gas effect. Energy at which wavelength is absorbed by the atmosphere and mainly causes this temperature increase?	Options for 1.:<br>A. Weather is a day-to-day event while climate is a consistent pattern over a year or longer.<br>B. Weather is local, but climate is global.<br>C. Weather is predicable but climate is not.<br>D. Weather includes more variables like moisture and wind while climate just focuses on temperature and precipitation.<br>E. I don't know.<br>For 2.:<br>A. carbon dioxide (CO_2)<br>B. water vapor (H_2O)<br>C. methane (CH_4)<br>D. oxygen (O_2)<br>E. ozone (O_3)<br>For 3.:<br>A. carbon dioxide (CO_2)<br>B. water vapor (H_2O)<br>C. methane(CH_4)<br>D. oxygen (O_2)<br>E. ozone (O_3)<br>For 4.:<br>A. radio<br>B. infrared<br>C. visible<br>D. ultraviolet<br>E. x-ray
**Rooney-Varga et al., 2018; Rooney-Varga et al., 2021**	6 items measuring impacts, urgency, hope, intent and values about climate change, for example:<br>Impacts of climate change—Increased temperatures globally<br>Impacts of climate change—Increased incidence and intensity of heat waves<br>Impacts of climate change—Increased rates of extinction of plant and animal<br>Impacts of climate change—Increased global sea level<br>Impacts of climate change—Increased intensity of storms across many regions<br>Impacts of climate change—an overall decrease in clean, potable water globally	Yes/No

**Note: studies by Vainio & Paloniemi (2013), Zhang et al. (2022), and Aruta (2023) measured only subjective knowledge about climate change.; Where the same or adapted measurement instrument was used, the items of separate studies are presented in one bracket.*
