# Supplementary material for: Measuring climate knowledge: A systematic review of quantitative studies
Source: iScience. 2025 Jan 25;28(2):111888. doi: 10.1016/j.isci.2025.111888 (PMC11869530; doi:10.1016/j.isci.2025.111888)
Supplement: List S4. List of studies in the sample [file mmc4.pdf]

List of studies in the final systematic review sample

Abunyewah, M., Erdiaw-Kwasie, M.O., Acheampong, A.O., Arhin, P., Okyere, S.A., Zanders, K., Frimpong, L.K., Byrne, M.K., and Lassa, J. (2023). Understanding climate change adaptation in Ghana: The role of climate change anxiety, experience, and knowledge. Environmental Science & Policy *150*, 103594. https://doi.org/10.1016/j.envsci.2023.103594.

Adu Boateng, E., Asibey, M.O., Cobbinah, P.B., Adutwum, I.O., and Blija, D.K. (2023). Enabling nature-based solutions: Innovating urban climate resilience. Journal of Environmental Management *332*, 117433. https://doi.org/10.1016/j.jenvman.2023.117433.

Alenda-Demoutiez, J. (2022). Climate change literacy in Africa: the main role of experiences. International Journal of Environmental Studies *79*, 981–997. https://doi.org/10.1080/00207233.2021.1987059.

Anyanwu, R., and Grange, L.L. (2017). The influence of teacher variables on climate change science literacy of Geography teachers in the Western Cape, South Africa. International Research in Geographical and Environmental Education *26*, 193–206. https://doi.org/10.1080/10382046.2017.1330039.

Aruta, J.J.B.R. (2023). Science literacy promotes energy conservation behaviors in Filipino youth via climate change knowledge efficacy: Evidence from PISA 2018. Australian Journal of Environmental Education *39*, 55–66. https://doi.org/10.1017/aee.2022.10.

Asgarizadeh, Z., Gifford, R., and Colborne, L. (2023). Predicting climate change anxiety. Journal of Environmental Psychology *90*, 102087. https://doi.org/10.1016/j.jenvp.2023.102087.

Asshoff, R., Konnemann, C., Tramowsky, N., and Rieß, W. (2021). Applying the Global Change App in Different Instruction Settings to Foster Climate Change Knowledge among Student Teachers. Sustainability *13*, 9208. https://doi.org/10.3390/su13169208.

Banwell, N., Gesche, A.S., Vilches, O.R., and Hostettler, S. (2020). Barriers to the implementation of international agreements on the ground: Climate change and resilience building in the Araucanía Region of Chile. International Journal of Disaster Risk Reduction *50*, 101703. https://doi.org/10.1016/j.ijdrr.2020.101703.

Bedford, D. (2016). Does Climate Literacy Matter? A Case Study of U.S. Students' Level of Concern about Anthropogenic Global Warming. Journal of Geography *115*, 187–197. https://doi.org/10.1080/00221341.2015.1105851.

Bodzin, A.M., Anastasio, D., Sahagian, D., Peffer, T., Dempsey, C., and Steelman, R. (2014). Investigating Climate Change Understandings of Urban Middle-Level Students. Journal of Geoscience Education *62*, 417–430. https://doi.org/10.5408/13-042.1.

Boon, H. (2016). Pre-Service Teachers and Climate Change: A Stalemate? Australian Journal of Teacher Education *41*. https://doi.org/10.14221/ajte.2016v41n4.3.

Borhan, M.T., and Ismail, Z. (2011). Pre-Service Teachers' Perception Toward Environmental Knowledge, Attitudes and Behaviours. Malaysian Journal of Learning and Instruction *8*, 117–137.

Bozoglu, M., Kilic Topuz, B., Başer, U., Shahbaz, P., and Alhas Eroğlu, N. (2022). Graduate Students' Knowledge Levels on Climate Change in the Departments of Agricultural Economics in Turkey. Journal of Agricultural Science and Technology *24*, 1029–1041.

Bremer, J., and Linnenluecke, M.K. (2017). Determinants of the perceived importance of organisational adaptation to climate change in the Australian energy industry. Australian Journal of Management *42*, 502–521. https://doi.org/10.1177/0312896216672273.

Carroll Steward, K., Gosselin, D., Chandler, M., and Forbes, C.T. (2024). Student Outcomes of Teaching About Socio-scientific Issues in Secondary Science Classrooms: Applications of EzGCM. J Sci Educ Technol *33*, 195–207. https://doi.org/10.1007/s10956-023-10068-7.

Chuvieco, E., Burgui-Burgui, M., Orellano, A., Otón, G., and Ruíz-Benito, P. (2021). Links between Climate Change Knowledge, Perception and Action: Impacts on Personal Carbon Footprint. Sustainability *13*, 8088. https://doi.org/10.3390/su13148088.

Connor, M., Cuong, O.Q., Demont, M., Sander, B.O., and Nelson, K. (2022). The influence of climate change knowledge on consumer valuation of sustainably produced rice in Vietnam. Sustainable Production and Consumption *31*, 1–12. https://doi.org/10.1016/j.spc.2022.01.034.

Das, U., Ansari, M.A., and Ghosh, S. (2022). Does climate knowledge act as a shield for farm livelihoods? Empirical analysis from the coastal and non-coastal ecosystems of India. Theor Appl Climatol *150*, 1627–1642. https://doi.org/10.1007/s00704-022-04245-8.

DeCamp, E. (2024). Integrating climate change across the disciplines: review of a faculty learning community and student climate literacy assessment model. Environmental Education Research *30*, 2159–2177. https://doi.org/10.1080/13504622.2024.2309588.

DeWaters, J., Andersen, C., Calderwood, A., and Powers, S. (2014). Improving Climate Literacy With Project-Based Modules Rich in Educational Rigor and Relevance. Journal of Geoscience Education *62*, 469–484. https://doi.org/10.5408/13-056.1.

Giusto, B.D., Lavallee, J.P., and Yu, T.-Y. (2018). Towards an East Asian model of climate change awareness: A questionnaire study among university students in Taiwan. PLOS ONE *13*, e0206298. https://doi.org/10.1371/journal.pone.0206298.

Dijkstra, E. m., and Goedhart, M. j. (2012). Development and validation of the ACSI: measuring students' science attitudes, pro-environmental behaviour, climate change attitudes and knowledge. Environmental Education Research *18*, 733–749. https://doi.org/10.1080/13504622.2012.662213.

Ebuehi, O.M., and Olusanya, O.A. (2013). Climate change: knowledge, attitude and practice among the residents of Ifo LGA, Ogun State, South West, Nigeria - challenges and prospects towards site-specific interventions. IJGW *5*, 345. https://doi.org/10.1504/IJGW.2013.055367.

Escoz Roldán, A., Arto-Blanco, M., Meira-Cartea, P.Á., and Gutiérrez-Pérez, J. (2019). Social Representations of Climate Change among Spanish University Students of the Social

Sciences and Humanities. The International Journal of Interdisciplinary Environmental Studies *13*, 1–14. https://doi.org/10.18848/2329-1621/CGP/v13i02/1-14.

Fernández, D.C., Gómez-Gonçalves, A., and Sánchez-Barbero, B. (2023). Effectiveness of Interdisciplinary Instruction in Pre-service Teacher Education for Sustainability: Issues From the Big History and the Study of Climate Change. Journal of Teacher Education for Sustainability *25*, 5–21. https://doi.org/10.2478/jtes-2023-0002.

Fischer, H., and Said, N. (2021). Importance of domain-specific metacognition for explaining beliefs about politicized science: The case of climate change. Cognition *208*, 104545. https://doi.org/10.1016/j.cognition.2020.104545.

Fischer, H., Amelung, D., and Said, N. (2019). The accuracy of German citizens' confidence in their climate change knowledge. Nat. Clim. Chang. *9*, 776–780. https://doi.org/10.1038/s41558-019-0563-0.

Flora, J., Saphir, M., Lappé, M., Roser-Renouf, C., Maibach, E., and Leiserowitz, A. (2014). Evaluation of a national high school entertainment education program: The Alliance for Climate Education. CLIMATIC CHANGE *127*, 419–434. https://doi.org/10.1007/s10584-014-1274-1.

García-Vinuesa, A., Carvalho, S., Meira Cartea, P.Á., and Azeiteiro, U.M. (2021). Assessing climate knowledge and perceptions among adolescents. An exploratory study in Portugal. The Journal of Educational Research *114*, 381–393. https://doi.org/10.1080/00220671.2021.1954582.

Gazzaz, N.M., and Aldeseet, B.A. (2021). Assessment of the Level of Knowledge of Climate Change of Undergraduate Science and Agriculture Students. World Journal of Education *11*, 41. https://doi.org/10.5430/wje.v11n5p41.

Geiger, S.M., Otto, S., and Diaz-Marin, J.S. (2014). A diagnostic Environmental Knowledge Scale for Latin America / Escala diagnóstica de conocimientos ambientales para Latinoamérica. PsyEcology *5*, 1–36. https://doi.org/10.1080/21711976.2014.881664.

Gutierrez, K.S., Blanchard, M.R., and Busch, K.C. (2022). What effective design strategies do rural, underserved students in STEM clubs value while learning about climate change? Environmental Education Research *28*, 1043–1069. https://doi.org/10.1080/13504622.2022.2032611.

Hallar, A.G., McCubbin, I.B., and Wright, J.M. (2011). CHANGE: A Place-Based Curriculum for Understanding Climate Change at Storm Peak Laboratory, Colorado. Bulletin of the American Meteorological Society *92*, 909–918. https://doi.org/10.1175/2011BAMS3026.1.

Harker-Schuch, I.E., Mills, F.P., Lade, S.J., and Colvin, R.M. (2020). CO2peration – Structuring a 3D interactive digital game to improve climate literacy in the 12-13-year-old age group. Computers & Education *144*, 103705. https://doi.org/10.1016/j.compedu.2019.103705.

Helbling, M., Auer, D., Meierrieks, D., Mistry, M., and Schaub, M. (2021). Climate change literacy and migration potential: micro-level evidence from Africa. Climatic Change *169*, 9. https://doi.org/10.1007/s10584-021-03241-7.

Higuchi, M.I.G., Paz, D.T., Roazzi, A., and Souza, B.C. de (2018). Knowledge and Beliefs about Climate Change and the Role of the Amazonian Forest among University and High School Students. Ecopsychology *10*, 106–116. https://doi.org/10.1089/eco.2017.0050.

Hu, S., Jia, X., Zhang, X., Zheng, X., and Zhu, J. (2017). How political ideology affects climate perception: Moderation effects of time orientation and knowledge. Resources, Conservation and Recycling *127*, 124–131. https://doi.org/10.1016/j.resconrec.2017.09.003.

Hurst Loo, A.M., and Walker, B.R. (2023). Climate change knowledge influences attitude to mitigation via efficacy beliefs. Risk Analysis *43*, 1162–1173. https://doi.org/10.1111/risa.14026.

Huxster, J.K., Uribe-Zarain, X., and Kempton, W. (2015). Undergraduate Understanding of Climate Change: The Influences of College Major and Environmental Group Membership on Survey Knowledge Scores. The Journal of Environmental Education *46*, 149–165. https://doi.org/10.1080/00958964.2015.1021661.

Jama, O.M., Diriye, A.W., and Abdi, A.M. (2023). Understanding young people’s perception toward forestation as a strategy to mitigate climate change in a post-conflict developing country. Environ Dev Sustain *25*, 4787–4811. https://doi.org/10.1007/s10668-022-02242-5.

Javeline, D., Kijewski-Correa, T., and Chesler, A. (2019). Does it matter if you “believe” in climate change? Not for coastal home vulnerability. Climatic Change *155*, 511–532. https://doi.org/10.1007/s10584-019-02513-7.

Jurek, M., Frajer, J., Fiedor, D., Brhelová, J., Hercik, J., Jáč, M., and Lehnert, M. (2022). Knowledge of global climate change among Czech students and its influence on their beliefs in the efficacy of mitigation action. Environmental Education Research *28*, 1126–1143. https://doi.org/10.1080/13504622.2022.2086687.

Karpudewan, M., and Mohd Ali Khan, N.S. (2017). Experiential-based climate change education: fostering students’ knowledge and motivation towards the environment. International Research in Geographical and Environmental Education *26*, 207–222. https://doi.org/10.1080/10382046.2017.1330037.

Karpudewan, M., Roth, W.-M., and Chandrakesan, K. (2015). Remediating misconception on climate change among secondary school students in Malaysia. Environmental Education Research *21*, 631–648. https://doi.org/10.1080/13504622.2014.891004.

Klapp, J., and Bouvier-Brown, N.C. (2021). Climate literacy among undergraduate students who study science in Los Angeles. International Journal of Sustainability in Higher Education *22*, 1707–1727. https://doi.org/10.1108/IJSHE-09-2020-0343.

Kolenatý, M., Kroufek, R., and Činčera, J. (2022). What Triggers Climate Action: The Impact of a Climate Change Education Program on Students’ Climate Literacy and Their Willingness to Act. Sustainability *14*, 10365. https://doi.org/10.3390/su141610365.

Kumar, P., Sahani, J., Rawat, N., Debele, S., Tiwari, A., Mendes Emygdio, A.P., Abhijith, K.V., Kukadia, V., Holmes, K., and Pfautsch, S. (2023). Using empirical science education in schools to improve climate change literacy. *Renewable and Sustainable Energy Reviews* 178, 113232. https://doi.org/10.1016/j.rser.2023.113232

Kurowski, L., Rutecka-Gora, J., and Smaga, P. (2022). Is knowledge on climate change a driver of consumer purchase decisions in Poland? The case of grocery goods and green banking. JOURNAL OF CLEANER PRODUCTION *369*. https://doi.org/10.1016/j.jclepro.2022.133444.

Liarakou, G., Athanasiadis, I., and Gavrilakis, C. (2011). What Greek Secondary School Students Believe about Climate Change? International Journal of Environmental and Science Education *6*, 79–98.

Lin, C.-H., and Wang, W.-C. (2023). Impacts of climate change knowledge on coastal tourists' destination decision-making and revisit intentions. Journal of Hospitality and Tourism Management *56*, 322–335. https://doi.org/10.1016/j.jhtm.2023.07.005.

Liu, Y., Song, Y., and Wang, X. (2022). Increasing Preservice Science Teachers' Climate Change Knowledge, Hope, and Self-Efficacy in an Online Chemistry Course. J. Chem. Educ. *99*, 2465–2473. https://doi.org/10.1021/acs.jchemed.2c00074.

Liu, Z., Smith, W.J., and Safi, A.S. (2014). Rancher and farmer perceptions of climate change in Nevada, USA. Climatic Change *122*, 313–327. https://doi.org/10.1007/s10584-013-0979-x.

McCright, A.M. (2010). The effects of gender on climate change knowledge and concern in the American public. Popul Environ *32*, 66–87. https://doi.org/10.1007/s11111-010-0113-1.

McNeill, K.L., and Vaughn, M.H. (2012). Urban High School Students' Critical Science Agency: Conceptual Understandings and Environmental Actions Around Climate Change. Res Sci Educ *42*, 373–399. https://doi.org/10.1007/s11165-010-9202-5.

Meira-Cartea, P.A., Gutiérrez-Pérez, J., Arto-Blanco, M., and Escoz-Roldán, A. (2018). Influence of academic education vs. common culture on the climate literacy of university students / Formación académica frente a cultura común en la alfabetización climática de estudiantes universitarios. PsyEcology *9*, 301–340. https://doi.org/10.1080/21711976.2018.1483569.

Mumpower, J.L., Liu, X., and Vedlitz, A. (2016). Predictors of the perceived risk of climate change and preferred resource levels for climate change management programs. Journal of Risk Research *19*, 798–809. https://doi.org/10.1080/13669877.2015.1043567.

Nepras, K., Strejckova, T., Kroufek, R., and Kubiatko, M. (2023). Climate Change Attitudes, Relationship To Nature And Pro-Environmental Behaviour Of Students From Three European Countries. Journal of Baltic Science Education *22*, Continuous. https://doi.org/10.33225/jbse/23.22.309.

Ngo, C.C., Poortvliet, P.M., and Feindt, P.H. (2020). Drivers of flood and climate change risk perceptions and intention to adapt: an explorative survey in coastal and delta Vietnam. Journal of Risk Research *23*, 424–446. https://doi.org/10.1080/13669877.2019.1591484.

Nussbaum, E.M., Owens, M.C., Sinatra, G.M., Rehmat, A.P., Cordova, J.R., Ahmad, S., Harris, F.C., and Dascalu, S.M. (2015). Losing the Lake: Simulations to Promote Gains in Student Knowledge and Interest about Climate Change. International Journal of Environmental and Science Education *10*, 789–811.

Nyarko, S.C., and Petcovic, H.L. (2021). Ghanaian preservice science teachers' knowledge of ozone depletion and climate change, and sources of their knowledge. International Journal of Science Education *43*, 1554–1575. https://doi.org/10.1080/09500693.2021.1922779.

Pan, W.-L., Fan, R., Pan, W., Ma, X., Hu, C., Fu, P., and Su, J. (2023). The role of climate literacy in individual response to climate change: evidence from China. Journal of Cleaner Production *405*, 136874. https://doi.org/10.1016/j.jclepro.2023.136874.

Peterson, G.H., and Kozlowski, M.B. (2024). Development and Initial Validation of the Climate Change Counseling Scale. *Measurement and Evaluation in Counseling and Development* 1–21. https://doi.org/10.1080/07481756.2024.2303461

Player, L., Hanel, P.H.P., Whitmarsh, L., and Shah, P. (2023). The 19-Item Environmental Knowledge Test (EKT-19): A short, psychometrically robust measure of environmental knowledge. Heliyon *9*. https://doi.org/10.1016/j.heliyon.2023.e17862.

Powers, S.E., DeWaters, J.E., and Dhaniyala, S. (2021). Climate Literacy—Imperative Competencies for Tomorrow's Engineers. Sustainability *13*, 9684. https://doi.org/10.3390/su13179684.

Rahman, M.S., Karamehic-Muratovic, A., Baghbanzadeh, M., Amrin, M., Zafar, S., Rahman, N.N., Shirina, S.U., and Haque, U. (2021). Climate change and dengue fever knowledge, attitudes and practices in Bangladesh: a social media–based cross-sectional survey. Transactions of The Royal Society of Tropical Medicine and Hygiene *115*, 85–93. https://doi.org/10.1093/trstmh/traa093.

Ratinen, I., and Uusiautti, S. (2020). Finnish Students' Knowledge of Climate Change Mitigation and Its Connection to Hope. SUSTAINABILITY *12*. https://doi.org/10.3390/su12062181.

Ratinen, I. (2021). Students' Knowledge of Climate Change, Mitigation and Adaptation in the Context of Constructive Hope. Education Sciences *11*, 103. https://doi.org/10.3390/educsci11030103.

Regassa, N., and Stoecker, B.J. (2014). Research Article: Attitude and Risk Perceptions about Climate Change in Farming Communities in Southern Ethiopia. Environmental Practice *16*, 29–36. https://doi.org/10.1017/S1466046613000628.

Acevedo, R.H.R., Reymundo, B.J.Q., Huamán, C.W.H., Orellana, J.C.Á., Berrocal, E.O., Ofracio, J.D.J., Velarde, Z.S., Quezada, U.R.Q., Salas, W.R., and Montalvan, C.E.Á. (2022). Knowledge, Attitude and Eco-Efficiency Urban Environment in the Inhabitants of the District of Chilca – Peru. Journal of Educational and Social Research *12*, 204. https://doi.org/10.36941/jesr-2022-0017.

Rooney-Varga, J.N., Hensel, M., McCarthy, C., McNeal, K., Norfles, N., Rath, K., Schnell, A.H., and Sterman, J.D. (2021). Building Consensus for Ambitious Climate Action Through

the World Climate Simulation. Earth's Future *9*, e2021EF002283. https://doi.org/10.1029/2021EF002283.

Rooney-Varga, J.N., Sterman, J.D., Fracassi, E., Franck, T., Kapmeier, F., Kurker, V., Johnston, E., Jones, A.P., and Rath, K. (2018). Combining role-play with interactive simulation to motivate informed climate action: Evidence from the World Climate simulation. PLOS ONE *13*, e0202877. https://doi.org/10.1371/journal.pone.0202877.

Schollaert Uz, S., Ackerman, W., O'Leary, J., Culbertson, B., Rowley, P., and Arkin, P.A. (2014). The Effectiveness of Science on a Sphere Stories to Improve Climate Literacy Among the General Public. Journal of Geoscience Education *62*, 485–494. https://doi.org/10.5408/13-075.1.

Seebauer, S. (2014). Validation of a social media quiz game as a measurement instrument for climate change knowledge. Entertainment Computing *5*, 425–437. https://doi.org/10.1016/j.entcom.2014.10.007.

Siegner, A., and Stapert, N. (2020). Climate change education in the humanities classroom: a case study of the Lowell school curriculum pilot. Environmental Education Research *26*, 511–531. https://doi.org/10.1080/13504622.2019.1607258.

Sorensen, A.E., Jordan, R.C., Blaise, G., Brown, J., Campbell, L.K., Aronson, M.F.J., and Johnson, M.L. (2018). Drivers of Public Participation in Urban Restoration Stewardship Programs: Linkages Between Environmental Identity and Knowledge, and Motivations. Arboriculture & Urban Forestry (AUF) *44*, 266–282. https://doi.org/10.48044/jauf.2018.024.

Stevenson, K.T., Peterson, M.N., Bondell, H.D., Moore, S.E., and Carrier, S.J. (2014). Overcoming skepticism with education: interacting influences of worldview and climate change knowledge on perceived climate change risk among adolescents. Climatic Change *126*, 293–304. https://doi.org/10.1007/s10584-014-1228-7.

Stevenson, K.T., Peterson, M.N., and Bradshaw, A. (2016). How Climate Change Beliefs among U.S. Teachers Do and Do Not Translate to Students. PLOS ONE *11*, e0161462. https://doi.org/10.1371/journal.pone.0161462.

Taddicken, M., Reif, A., and Hoppe, I. (2018). What do people know about climate change — and how confident are they? On measurements and analyses of science related knowledge. JCOM *17*, A01. https://doi.org/10.22323/2.17030201.

Thacker, I. (2023). Climate change by the numbers: Leveraging mathematical skills for science learning online. Learning and Instruction *86*, 101782. https://doi.org/10.1016/j.learninstruc.2023.101782.

Thaller, A., and Brudermann, T. (2020). "You know nothing, John Doe" – Judgmental overconfidence in lay climate knowledge. Journal of Environmental Psychology *69*, 101427. https://doi.org/10.1016/j.jenvp.2020.101427.

Tolppanen, S., Kang, J., and Tirri, K. (2023). Climate Competencies of Finnish Gifted and Average-Ability High School Students. Education Sciences *13*, 840. https://doi.org/10.3390/educsci13080840.

Tranter, B. (2020). Does Public Knowledge of Climate Change Really Matter in Australia?*. Environmental Communication *14,* 537–554. https://doi.org/10.1080/17524032.2019.1696853.

Tranter, B. (2021). Climate Change Knowledge and Political Identity in Australia. Sage Open *11*, 21582440211032673. https://doi.org/10.1177/21582440211032673.

Tranter, B., Skrbiš, Z., and Smith, J.F. (2020). Poles Apart: Political Divisions over Climate Change Among Younger Australians. JAYS *3,* 255–273. https://doi.org/10.1007/s43151-020-00013-5.

Trémolière, B., and Djeriouat, H. (2021). Exploring the roles of analytic cognitive style, climate science literacy, illusion of knowledge, and political orientation in climate change skepticism. Journal of Environmental Psychology *74,* 101561. https://doi.org/10.1016/j.jenvp.2021.101561.

Vainio, A., and Paloniemi, R. (2013). Does belief matter in climate change action? Public Underst Sci *22*, 382–395. https://doi.org/10.1177/0963662511410268.

Walker, S.L., and McNeal, K.S. (2013). Development and Validation of an Instrument for Assessing Climate Change Knowledge and Perceptions: The Climate Stewardship Survey (CSS). International Electronic Journal of Environmental Education *3*, 57–73.

Wang, Y., Zhang, X., Li, Y., Liu, Y., Sun, B., Wang, Y., Zhou, Z., Zheng, L., Zhang, L., Yao, X., et al. (2022). Knowledge, Attitude, Risk Perception, and Health-Related Adaptive Behavior of Primary School Children towards Climate Change: A Cross-Sectional Study in China. International Journal of Environmental Research and Public Health *19,* 15648. https://doi.org/10.3390/ijerph192315648.

Wang, H.-H., Bhattacharya, D., and Nelson, B.J. (2020). Secondary agriculture teachers' knowledge, beliefs and teaching practices of climate change. *The Journal of Agricultural Education and Extension* 26, 5–17. https://doi.org/10.1080/1389224X.2019.1699126

Wodika, A., and Schoof, J. (2017). Assessing climate change education on a Midwestern college campus. Applied Environmental Education & Communication *16*, 262–275. https://doi.org/10.1080/1533015X.2017.1348268.

Yeh, S.-C., Chen, Y.-H., Van Velzen, R., and Lin, P.-H. (2024). The climate change literacy of public officials in Taiwan: implications and strategies for global adaptation. Policy Studies *0,* 1–34. https://doi.org/10.1080/01442872.2024.2304086.

Zhang, J., Tong, Z., Ji, Z., Gong, Y., and Sun, Y. (2022). Effects of Climate Change Knowledge on Adolescents' Attitudes and Willingness to Participate in Carbon Neutrality Education. International Journal of Environmental Research and Public Health *19*, 10655. https://doi.org/10.3390/ijerph191710655.
